# Targeting APE1/Ref-1 to alleviate formalin-induced pain and spinal neuro-inflammation in rats: a promising therapeutic approach

**DOI:** 10.3389/fnins.2025.1542264

**Published:** 2025-07-30

**Authors:** Eman Elgendy, Amira Zaky, Mayssaa Wahby, Marc Landry, Ahmad Bassiouny

**Affiliations:** ^1^Department of Biochemistry, Faculty of Science, Alexandria University, Alexandria, Egypt; ^2^University of Bordeaux, CNRS, Institute of Neurodegenerative Diseases, IMN, UMR 5293, Bordeaux, France

**Keywords:** apurinic/apyrimidinic endonuclease 1 (APE-1/Ref-1), spinal cord, nociception, E3330 [(2E)-2-[(4,5-dimethoxy-2-methyl-3,6-dioxo-1,4-cyclohexadien-1-yl) methylene]-undecanoic acid, formalin (FA), dopamine, dopamine receptors, inflammasomes

## Abstract

**Background:**

Pain is a multifaceted condition intricately linked to inflammation, which plays a critical role in its onset and progression.

**Methods:**

To investigate the influence of APE1/Ref-1 on oxidative stress and inflammatory marker expression, we employed a hind paw sensitization model induced by formalin. We inhibited the redox function of APE1 using E3330 and assessed its effects on pain behavior. Mitochondrial morphology was examined via electron microscopy, and the impact on dopaminergic signaling alongside bioinformatics analyses to explore potential E3330 binding to dopamine receptors.

**Results:**

Administration of E3330 in formalin-induced rats resulted in improved pain thresholds, as evidenced by behavioral assessments. Notably, E3330 treatment maintained normal APE1/Ref-1 levels and promoted a more organized mitochondrial structure. Administration of E3330 correlated with increased dopamine levels, a decrease in the mRNA expression of dopamine receptors DRD1 and DRD5, and a restoration of DRD2 expression in the ipsilateral spinal cords. Moreover, E3330 administration significantly reduced the expression of key inflammatory mediators including inflammasome markers. Our bioinformatics analysis using Molecular Operating Environment software indicated that E3330 possibly interacts with critical active sites within specific dopamine receptor pocket as preliminary results.

**Conclusion:**

These findings suggest that E3330 may modulate pain signaling pathways from the periphery to the spinal cord, offering a novel approach for the management of inflammatory pain conditions, potentially through the modulation of the dopaminergic signaling pathway. Further research is warranted to elucidate E3330’s role in regulating central nervous system pain signal transmission, as it emerges as a promising therapeutic candidate in clinical contexts.

## 1 Introduction

Pain sensation is a complex experience that engages a network of pathways extending from peripheral nerves to the spinal cord and higher brain centers (Vardeh et al., 2016). Inflammatory conditions often involve intricate interactions between nociceptors and various immunomodulatory components, which serve to amplify nociceptive inputs throughout the central nervous system (Yam et al., 2020).

Animal models have successfully replicated inflammatory pain through the intra-paw injection of formalin (Fu et al., 2001). This agent induces local inflammation by instigating the release of a wide array of inflammatory mediators from resident immune cells, leading to significant alterations at peripheral nerve terminals (Clavelou et al., 1995). The resultant increase in nociceptive signaling to the spinal cord, coupled with the activation of spinal microglia, promotes central sensitization and long-lasting hyperalgesia (Zhang et al., 2018) a condition increasingly linked to elevated levels of reactive oxygen species (ROS) (Persoz et al., 2010). The presence of inflammation is often accompanied by oxidative stress, with well-documented interactions between oxidative stress and nociceptive pathways (Hendrix, 2020; Herzberg et al., 2019). Notably, ROS, particularly those generated within the mitochondria, can facilitate the activation of nuclear factor kappa-B (NF-κB) (Morgan and Liu, 2011). Moreover, researchers highlighted the strong implication of the inflammasomes which are innate immune system receptors/sensors (Dawson and Jenkins, 2024). The inflammasome signaling regulates the activation of caspase-1 and induce inflammation in response to inflammatory disorders (Zheng et al., 2020). Inflammasome activation, especially the NLRP3 inflammasome, plays a key role in inflammatory pain by triggering pro-inflammatory cytokine release, which heightens pain sensitivity and mitochondrial damage (Starobova et al., 2020). Although Formalin injection into the hind paw is a well-established model for inducing inflammatory pain but its direct activation of the NLRP3 inflammasome is still being explored.

Apurinic/apyrimidinic endonuclease 1 (APE1, also referred to as Ref-1 or APEX1) functions as a redox sensor protein with multifaceted regulatory roles. It is integral to DNA repair processes, transcriptional regulation, and redox control mechanisms (Cesaratto et al., 2013). APE1 has garnered attention as a potential therapeutic target in the contexts of cancer and inflammation (Jedinak et al., 2011). While it is not directly connected to pain sensation, APE1 influences cellular mechanisms that can indirectly modulate pain perception (Park et al., 2013). Notably, its regulation of redox activity has been implicated in pain responses, particularly in models of inflammatory pain, such as that induced by complete Freund’s adjuvant in rodents (Zaky et al., 2018). The role of redox signaling is critical for modulating a range of transcription factors and is closely linked to the regulation of genes associated with inflammation and pain, including NF-κB (Tell et al., 2009). Additionally, an analysis report by Tang et al. (2021) indicated that APE1/Ref-1 can modulate NLRP3 activity indirectly through its redox regulation of NF-κB. E3330, a potent and selective inhibitor of APE1’s redox domain, spares the base excision repair function, thereby effectively inhibiting APE1’s redox activity. This action prevents the reduction of NF-κB, consequently exerting notable anti-inflammatory effects (Jedinak et al., 2011).

Dopamine (DA) serves a multifaceted role in the modulation of pain, influencing nociceptive signaling, the processing pathways of pain, and the emotional and motivational dimensions associated with pain. It is implicated in the transition from acute to chronic pain and possesses reported immunomodulatory properties (Li et al., 2022). All subtypes of dopamine receptors are expressed in primary nociceptors and various layers of the spinal cord’s dorsal horn (Puopolo, 2019). Among these, the DRD2 receptor is particularly notable for its involvement in both presynaptic and postsynaptic modulation of pain signals (Puopolo, 2019).

The present study aims to investigate the selective redox activity of APE1 within a formalin-induced inflammatory pain model, elucidating the protective characteristics of E3330 against inflammation and pain sensitization. Additionally, this research explores the potential of targeting dopaminergic signaling pathways in the spinal cord as a novel strategy for mitigating inflammatory pain.

## 2 Materials and methods

### 2.1 Experimental design

**Figure 1:**
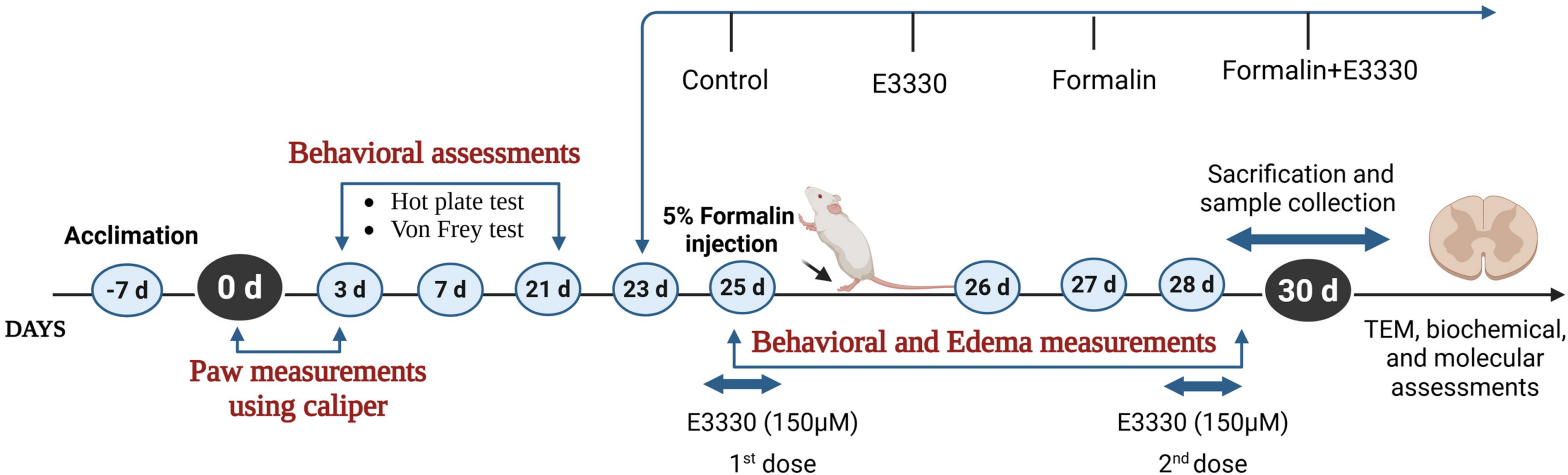
Experimental design timeline.

Sixty adult male Sprague–Dawley rats (250-300 g) were housed at the animal house of the Medical Research Institute, Alexandria University and used in this study. The sample size was determined using G*power 3.1.9.7. Priori power analysis was conducted to test differences between groups, using one-way ANOVA with effect size (*f* = 0.5), and alpha = 0.05 and we increased the number to avoid mortality if any. Animal experiments complied with the ARRIVE guidelines in accordance with EU Directive 2010/63/EU for animal experiments. The rats were housed as five rats per cage (187.88 length × 91.44 width × 17.8 height) under pathogen-free conditions with a 12 h light–dark cycle. They had continuous access to food and water throughout the study period. The animals were allowed to acclimate to laboratory conditions for 1 week before the experiment began. After the acclimatization period, the rats were evenly divided into four groups, with 14 rats in each group: a control group, E3330 group, formalin-induced group, and a formalin+E3330 administered group (Figure 1). The animal experimental protocol, including the justification for the use of animals, housing, feeding, environmental conditions as well as the mode of anesthesia were verified and approved by the Institutional Animal Care and Use Committee (IACUC) at Alexandria University (ethical approval No. AU04190629102), in accordance with the guidelines of the International Association for the Study of Pain.

### 2.2 Pain induction protocol

Inflammatory pain was induced by injecting 50 μL of 5% formalin (BDH Laboratory suppliers) subcutaneously into the dorsal surface of the left hind paw of the rats. Control animals received 50 μL of saline solution (NaCl 0.9%). Animals of the E3330 group were injected twice (at day 25 and day 28) subcutaneously with150 μM of E3330 (Sigma-Aldrich, n°E8534) suspended in NaCl and the formalin+E3330 group received 150 μM of E3330 twice (first; at day 25 1 hour before formalin induction and the second injection at day 28).

E3330 exhibits a half-life of approximately 3.6 to 5.6 h. Rat pharmacokinetic studies report similar elimination kinetics, supporting dosing intervals that allow sustained drug levels without rapid clearance (Luo et al., 2012).

For Tissue Distribution, studies, including those in rats, confirm that E3330 penetrates key target tissues such as the nervous system and retina, which is critical for its neuroprotective and anti-inflammatory actions. Also, both mice and rats tolerate E3330 well at doses up to 75 mg/kg, with no significant toxicity observed, supporting the safety of the dosing regimens used in preclinical efficacy studies (Heisel et al., 2021).

The two-dose regimen of E3330 (Day 25 and Day 28) is strategically aligned with the formalin-induced inflammatory timeline. The first dose targets early-phase inflammatory signaling, while the second dose maintains drug exposure during late-phase pathology and immediately prior to tissue collection (Fu et al., 2001; Liu et al., 2019). Given the short half-life of E3330 in rodents, this two-dose design enhances the likelihood of observing both behavioral and molecular effects attributable to Ref-1 inhibition.

The first dose of E3330 (150 μM) is given immediately after formalin injection (day 25), targeting the acute phase of inflammation and pain sensitization when molecular and cellular changes are most pronounced. Also, Giving the first dose on Day 25 allows for immediate pharmacological intervention during the initiation phase of inflammation and oxidative stress a key period where APE1/Ref-1 inhibition (E3330’s mechanism) may modulate early molecular responses (e.g., NF-κB activation, ROS signaling, pro-inflammatory cytokine release).

The second dose on day 28 is timed to coincide with the subacute/chronic phase of the inflammatory response (Li et al., 2010). This allows assessment of E3330’s effects on both the initial and sustained phases of pain and inflammation, mimicking clinical scenarios where repeated dosing may be necessary for ongoing symptoms.

### 2.3 Assessment of physical and behavioral parameters

#### 2.3.1 Measurement of paw edema

It is the most used experimental method for evaluating the induction of inflammation in a rat model, representing the early phase of inflammation. Formalin-induced paw edema was assessed by measuring the height of the injected paws as described by Singh et al. (2010) method using a Vernier caliper (SMEC, Shanghai, China). The paw edema height was measured on day 0 (before injection) then on days 1, 2, 3, 4, and finally on day 5 after formalin injection and before animal sacrifice. Results were expressed as percentage change relative to the control group.

#### 2.3.2 The hot plate test

The hot plate test was done according to the method of Minett et al. (2011) to measure animal nociceptive response latencies to thermal stimulus. Briefly, animals were placed individually on a hot plate (Ugo Basile, Varese, Italy) with the temperature adjusted to 55°C. The paw withdrawal latencies represent the time for the rat to respond to the pain sensitization associated with the heat. The time was recorded between the placement of the rat on the plate and the first sign of a nociceptive response such as paw licking or jumping. A cut-off time of 30 s was used to avoid heat-induced tissue damage to the hind paws. The paw withdrawal latency was calculated as percentage differences in comparison to the control group.

#### 2.3.3 The von Frey test

The von Frey test was conducted to quantify mechanical thresholds in unrestrained animals, following the methodology established by Maximilian von Frey, with modifications to better simulate the original electrical model (Minett et al., 2011; Deuis et al., 2017). The testing commenced with a calibrated probe featuring a diameter of 1.33 mm, measured using a micrometer screw gauge. Various hooked weights with masses of 0.05, 0.1, 0.15, 0.2, and 0.25 kg were used.

To determine the corresponding force (F) exerted by each weight, we utilized the formula F = mg, where F is the force, m is the mass of the hooked weight, and g is the acceleration due to gravity, which is approximately 9.8 m/s^2^. The specific distances associated with each force measurement were established, as detailed in Table 1, based on the displacement resulting from the application of each hooked weight. Subsequently, we recorded the paw withdrawal threshold of the rats in response to mechanical stimuli.

**Table 1:** Applied forces and corresponding distances for mechanical stimulation.

Force (*N*)	Distance (cm)
0.49	2
0.981	3.2
1.47	4.2
1.962	5.3
2.4525	6

The rats were individually housed in a testing area (mesh cage) for a minimum acclimation period of 25 min to facilitate ambulation and exploratory behaviors, thus reducing the risk of misinterpreting such behaviors as positive responses to nociceptive stimuli. A positive response was classified when the animal exhibited nocifensive behaviors, such as rapid withdrawal of the paw or licking and shaking of the affected paw during the application of the mechanical stimulus. The paw withdrawal threshold in response to mechanical stimulation was assessed before, 1 hour after, and on day four following the formalin injection. The results were expressed as percentage changes in the withdrawal thresholds across all experimental groups.

#### 2.3.4 Sample collection and tissue preparation

Sample collections were performed 5 days post-induction. Animals were allowed to fast for 12 h then a quiet environment was used to prepare the rats for anesthesia. Rats were placed in the anesthesia induction chamber and the isoflurane anesthetic gas was introduced at a concentration of 3% to induce anesthesia quickly with a calibrated flow meter to ensure accurate anesthetic gas delivery (Oh and Narver, 2024). Once anesthesia was induced, the concentration was lowered to 1% for maintenance during the procedure. Blood samples were collected from the inferior vena cava of all groups in sterilized plain Wassermann tubes, allowed to stand for 15 min at room temperature. Then samples were centrifuged at 4°C and 5,000 rpm using a PLC-05 series centrifuge for 10 min. The collected sera were divided into aliquots and kept at −80°C until used for analysis. Animals were sacrificed by decapitation then the lumbar spinal cord tissues were quickly removed. The ipsilateral side of the spinal cord was separated from the contralateral side with a scalpel. Spinal cord tissues were snap-frozen and stored at −80°C for future molecular and biochemical analyses. In parallel, another sampling was applied on some spinal cord tissues for imaging analysis as described below using transmission electron microscopy.

#### 2.3.5 Transmission electron microscopy

Rat spinal cord specimens were prepared according to Karnovsky et al. (1965) method for using transmission electron microscopy. Following perfusion with 4% formaldehyde and 1% glutaraldehyde, the rat spinal cord was removed quickly and evenly then the lumbar region was cut and immersed immediately in 4% formaldehyde and 1% glutaraldehyde in phosphate buffer pH 7.2 at 4°C. Specimens were placed in 2% osmium tetroxide (OsO4) in acetone at a temperature of −60 to −70°C. Then, the samples were dehydrated and passed through a “transition solvent” such as propylene oxide, infiltrated, warmed then embedded in a liquid resin, epoxy, and LR white resin. Ultrathin sections (50–70 nm) were obtained by ultramicrotomy, collected on metal mesh “grids”, and subject to electron-dense staining with uranyl acetate and lead citrate for 5 min per stain before examination in the TEM. This examination was conducted at the Electron Microscope Unit within the Faculty of Science at Alexandria University.

### 2.4 Oxidative stress-related markers

#### 2.4.1 Reduced glutathione assay

Dissected ipsilateral spinal cord tissues (10% w/v) were washed with PBS solution and then minced and homogenized in ice-cooled buffer (50 mM potassium phosphate buffer pH7.5 and 1 mM EDTA). The homogenates were centrifuged at 4000 rpm for 20 min at 4°C and the supernatants were used for determination of glutathione (GSH) and protein contents. The GSH level was determined according to the method of Beutler et al. (1963) and expressed as mmol/g tissue. Total protein level was determined according to the method of Gornall et al. (1949) and expressed as (g/dl).

#### 2.4.2 Malondialdehyde assay

Spinal cord tissues (10% w/v) were washed with PBS solution, minced, and homogenized in ice-cooled buffer (50 mM potassium phosphate, pH = 7.5.). The homogenates were centrifuged at 4,000 rpm for 15 min at 4°C. The supernatants were used for the MDA assay according to Ohkawa et al. (1979) colorimetric method. MDA levels were expressed as nmol/g tissue.

#### 2.4.3 Catalase activity assay

Catalase activity was determined according to the method of Aebi (1984) in the spinal cord tissue. Spinal cord tissues were homogenized in ice-cooled homogenization buffer (50 mM potassium phosphate, pH = 7.4, 1 mM EDTA, and 1 mL/L Triton X-100). The homogenate was centrifuged at 4,000 rpm for 15 min at 4°C and the supernatants were used for the assay. Catalase activity was expressed in μmol/min//g. tissue.

#### 2.4.4 Nitric oxide assay

Serum and spinal cord levels of nitric oxide were determined according to Montgomery and Dymock (1962) method. The spinal cord samples were homogenized in a cold buffer (100 mM potassium phosphate pH = 7 containing 2 mM EDTA). The homogenates were centrifuged at 4,000 rpm for 15 min at 4°C and the supernatants were used for the assay. Nitric oxide level was expressed in μmol/l and μmol/l/g. Protein for sera and tissues, respectively.

#### 2.4.5 DPPH (2,2-diphenylr-1-picrylhydrozyl) radical scavenging assay

The percentage of free radical scavenging in the spinal cord was determined according to the method of Shimamura et al. (2014) with some modifications and proper standardization. The spinal cord tissue was homogenized in 80% methanol and the homogenate vibrated for 30 min at room temperature and then centrifuged for 15 min at 4000 rpm. Fifty microliters of the supernatant were added to 1 mL solution of DPPH and kept in the dark. The capacity of a reaction solution was measured at 517 nm in a time-dependent manner; 3 min, 15 min, and 30 min, respectively. The radical Scavenging efficiency of the sample was calculated as follows:


DPPH Scavenging%=[(Acontrol–Asample)/Acontrol]100%

A control means the absorbance of the control (DPPH solution without the sample).

A sample means the absorbance of the sample (DPPH solution plus the sample).

Though the most standard results were obtained at the three-minute measurement of the redox scavenging activity using DPPH assay as presented in the result section, the other time courses were exponentially related to the 3 min time interval.

### 2.5 Molecular assessments

#### 2.5.1 RNA extraction and quantitative real-time PCR

RNA was extracted from the spinal cord as described by Chomczynski (1993) using Triazol (Invitrogen, Carlsbad, CA, n°15,596,026). Complementary DNA (cDNA) was synthesized using the power cDNA synthesis kit (Intron Biotechnology, Cat. No. 25011) to convert total mRNA into cDNA. Quantitative real-time PCR was applied to amplify the target genes using specific primer sets (listed in Table 2) in the presence of the SensiFAST™ SYBR^®^ No-ROX One-Step Kit (BIO-65053). Then, sample tubes were inserted into the thermal cycler device (QIAGEN RT-PCR system).

**Table 2:** The sequence of primers used in q-RT PCR.

Primer name	Sequence	Accession number
Beta-Actin (Actb)	Forward: AGC CAT GTA CGT AGC CAT CC	NM_031144
	Reverse: CTC TCA GCT GTG GTG GTG AA	
APE-1, apurinic/apyrimidinic endo deoxyribonuclease 1 (Apex1)	Forward: TGG AAT GTG GAT GGG CTT CGA GCC	NM_024148
	Reverse: AAG GAG CTG ACC AGT ATT GAT GA	
Nuclear factor kappa B subunit p65 (NF-κB)	Forward: ACG ATC TGT TTC CCC TCA TCT	AF079314.2
	Reverse: TGC TTC TCT CCC CAG GAA TA	
Interleukin 6 (IL-6)	Forward: AGT TGC CTT CTT GGG ACT GA	M26744
	Reverse: ACA GTG CAT CAT CGC TGT TC	
Inducible nitric oxide synthase (iNOS)	Forward: AAT GAA CCA CCC GAC TGA AG	AB250951
	Reverse: TTA TAC ACG GAA GGG CCA AG	
Tumor necrosis factor (TNF-α)	Forward: AGA TGT GGA ACT GGC AGA GG	L00981
	Reverse: CCC ATT TGG GAA CTT CTC CT	
Tumor necrosis factor receptor 1 (TNFR1)	Forward: ACC GGA CTG GTT CCT TCT CT	AF329980
	Reverse: CAC ACA CCT CGC AGA CTG TT	
NLR family pyrin domain containing 3 (NLRP3)	Forward: CTCGCATTGGTTCTGAGCTC	NM_001191642.1
	Reverse: AGTAAGGCCGGAATTCACCA	
Caspase-1	Forward: ACAAAGAAGGTGGCGCATTT	NM_012762.3
	Reverse: AACATCAGCTCCGACTCTCC	
Interleukin 1 beta (IL-1β)	Forward: GCA ATG GTC GGG ACA TAG TT	NM_031512
	Reverse: AGA CCT GAC TTG GCA GAG GA	
Interleukin 10 (IL-10)	Forward: GGG AAG CAA CTG AAA CTT CG	X60675
	Reverse: ATC ATG GAA GGA GCA ACC TG	
Brain-derived neurotrophic factor (BDNF)	Forward: GCG GCA GAT AAA AAG ACT GC	AY176065
	Reverse: GTA GTT CGG CAT TGC GAG TT	
Tropomyosin receptor kinase B (TrkB)	Forward: TTGTCCATTCCCTCACCCTC	AY265419.1
	Reverse: ACACGCTGGGACTGTTAAGA	
Glial cell-derived neurotrophic factor (GDNF)	Forward: GCC GAG ACA ATG TAC GAC AA	NM_019139
	Reverse: CTG GAG CCA GGG TCA GAT AC	
Dopamine receptor D5 (DRD5)	Forward: GCA AGG CTG GGA TTA CAG AG	NM_012768
	Reverse: ATG GCA GCA CAC ACT AGC AC	
Dopamine receptor D2 (DRD2)	Forward: ATC CAC TGA ACC TGT CCT GG	NM_012547.2
	Reverse: GTA GTT GTA GTG GGG CCT GT	
Dopamine receptor D1 (DRD1)	Forward: TCGAACTGTATGGTGCCCTT	NM_012546.3
	Reverse: AAGAATTCGCCCACCCAAAC	

#### 2.5.2 Enzyme-linked immunosorbent assay measurement

The quantitative determination of APE1, NF-κB, and IL-6 protein levels in rat spinal cord tissues were done using commercially available Rat ELISA kits according to the manufacturer guidelines. The kits were obtained from SinoGeneclon Co., Ltd. company supplier with Catalog No: SG-21531, SG-20807, and SG-20267, respectively.

#### 2.5.3 HPLC-PDA quantitative determination of dopamine level

The serum level of dopamine was determined according to the method of Yang and Beal (2011) and Gu et al. (2015) using high-performance liquid chromatography (Agilent Technologies^®^ Model 1,260 Infinity) with UV-photodiode array detector (HPLC-PDA). The equipment was accessed at the Scientific Academy for Pharmaceutical Research Unit within the Faculty of Pharmacy, Alexandria University. After the preparation of the sera, 50 μL of the supernatant was injected in HPLC for running according to the following chromatographic conditions: C18 column, 5 μm particles, size 150 cm × 4.6 mm I. D, mobile phase 5 mM HClO4 solution with 5% acetonitrile at flow rate 0.5 mL/min and 25°C using UV-PDA detector. The UV-PDA detection was conducted at wavelengths of 210 nm and 280 nm. The method has been validated for DA standard within the range of 10–100 μg/mL (r2 = 0.9933). Serum dopamine concentration was calculated using the standard equation (y = 1.4723x−8.9297), which was measured by using the calibration curve of the DA standard. The results of the experimental sera sample analysis relied on the detection of the standard DA peak at wavelength 210 nm, which is associated with the detection of catecholamine. DA identification was confirmed using a spectrum at a wavelength of 280 nm, a validated peak for DA release (Chen et al., 2021).

### 2.6 Docking method; an in-silico approach

#### 2.6.1 Retrieval of target protein sequence from the database

The amino acid sequence of DRD1, DRD5, and DRD2 dopamine receptors (ID: P18901, ID: P25115, and ID: P61169, respectively) of *Rattus norvegicus* was obtained from the UniProt database.^1^

#### 2.6.2 Template searching

We encountered difficulty in finding the co-crystalized structure of rat DA receptor in the protein database bank (PDB). Hence, to overcome such an obstacle, we worked through the application of a Swiss modeling server to predict the 3D structure with PDB format as referenced by Waterhouse et al. (2018), we selected the structure of DA receptors with high sequence identity compared to the DA receptors configuration available in the widely used protein database bank (PDB).

#### 2.6.3 Molecular docking protocol

Docking experiments of active proteins were performed via Molecular Operating Environment (MOE 2014.13) MOE is a chemical Computing Group Inc., software Docking that provides a reasonable explanation for the structure–activity relationships. We aim to explore the possible binding modes of the receptors with E3330 into the active sites to justify our experimental findings in part. The PDB structures of DRD1, DRD5, and DRD2 were prepared by eliminating unwanted residues, ligands, and water molecules, and then subjected to the default “structure preparation” module settings; where hydrogen atoms were added, hydrogen bonds were optimized, and atomic clashes were removed. The protein structures and compounds (DA and E3330) under investigation were built in silico and energy was minimized utilizing the MMFF94x force field at a gradient of 0.01 RMSD and optimized using the default MOE settings, the docking protocol was conducted by employing the triangular matcher algorithm as the ligand placement method and London dG as the scoring function, generating the top 10 nonredundant poses of the conformers with the lowest binding energies. The active site was selected based on the works of literature and the co-crystallized ligand interactions taking into consideration the reported binding sites. Among the top-ranked poses according to docking scores, molecular interactions, and RMSD values, the ligand-interaction module of MOE was used to calculate the 2D and 3D receptor-compound interactions. The view of the docking results and analysis of their surface with graphical representations were done using MOE.

### 2.7 Statistical analysis

The data were statistically analyzed according to the method of Muenchen (2011). The experimental results and graphs represented the mean and the standard errors of the mean (mean ± SEM). The differences between groups were analyzed by one-way analysis of variance (ANOVA) using IBM SPSS software program version 16.0 (Chicago, United States). The statistical difference between the experimental groups was analyzed by one-way analysis of variance (ANOVA) with Tukey’s honestly significant difference (HSD) *post hoc* test and independent sample *t*-test. The difference is significant at *p* < 0.05, *p* < 0.01, or *p* < 0.001. The detailed parameters of statistical analysis are listed in Supplementary Table S1.

## 3 Results

### 3.1 Formalin induces peripheral inflammation, paw edema, and pain hypersensitivity

**Figure 2:**
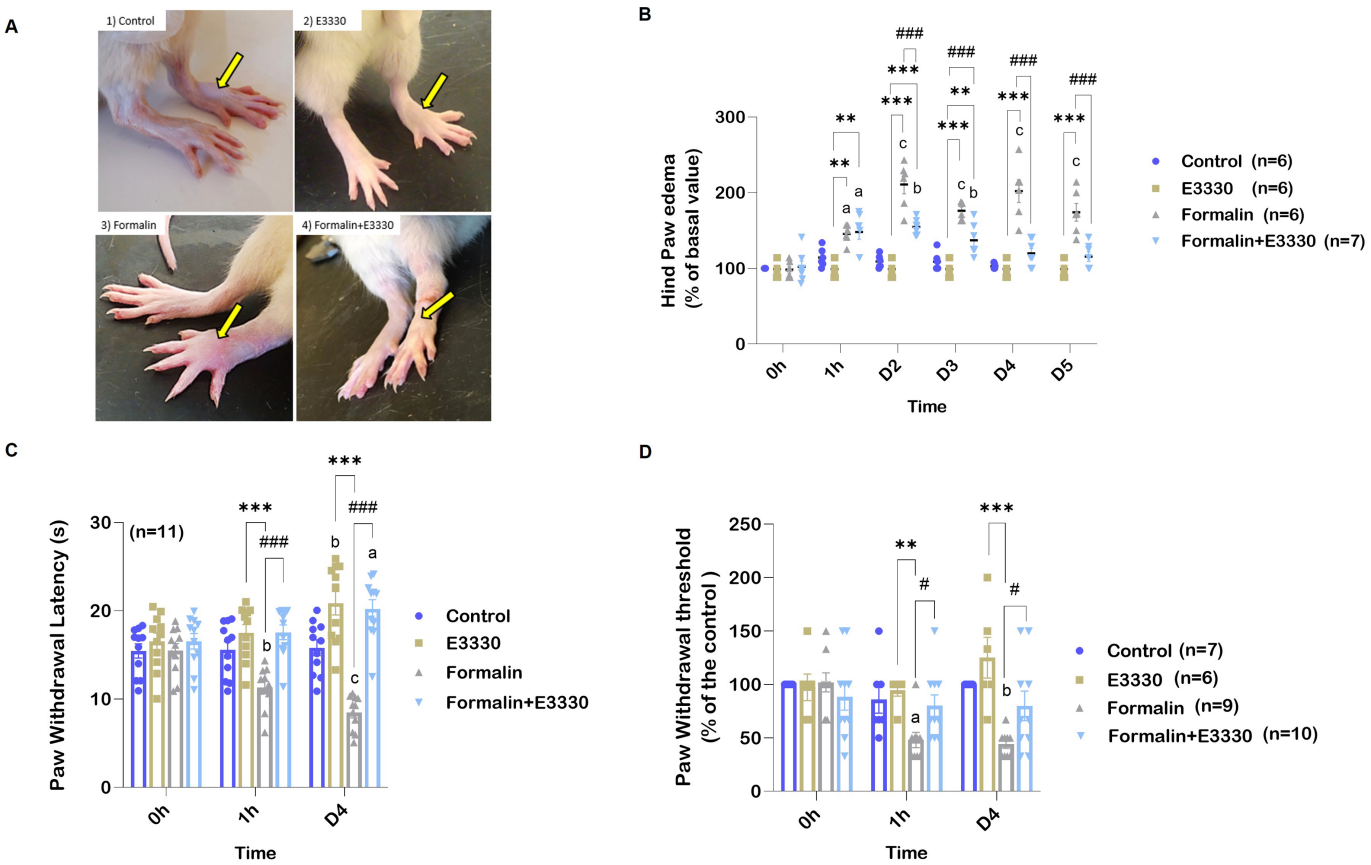
E3330 mitigates paw edema and pain sensitization that follows formalin-induction. **(A)** Photographs represent the alteration in the hind paw volume. **(B)** Percentage Changes in the examined rats’ hind paw edema. **(C)** Changes in paw withdrawal threshold of the different experimental groups after thermal stimulus. **(D)** Changes in paw withdrawal threshold of the different experimental groups after mechanical stimulus. Data represent the mean ± SEM; the significance versus control, E3330, and formalin are: ^a,*,#^ *p* < 0.05, ^b,**,##^ *p* < 0.01, ^c,***,###^ *p* < 0.001, respectively.

The administration of formalin-induced rapid peripheral inflammation, evidenced by the onset of paw edema on the injected side within 5 min following subcutaneous injection. Paw edema served as an indicator of local inflammation resulting from the formalin injection, assessed by measuring the size of the ipsilateral hind paw (Figures 2A,B; Supplementary Dataset S1). Notably, formalin injection resulted in a significant increase in hind paw height (*p* < 0.01 and *p* < 0.001, respectively) at 1 hour and on day two post-injection, with inflammation persisting until the day of dissection when compared to the control and E3330 groups.

Conversely, the formalin+E3330 group exhibited a significant reduction in hind paw distention (*p* < 0.001) on day two post-formalin injection compared to the formalin group. Additionally, 1 hour after formalin induction, the formalin+E3330 group demonstrated a significant increase (*p* < 0.01) in hind paw volume compared to both the control and E3330 groups; however, this increase returned to baseline levels by days three and four (*p* < 0.01; *p* < 0.05, respectively).

Beyond the acute effects of formalin, this irritant is known to induce prolonged secondary thermal and mechanical hyperalgesia. Specifically, formalin (5%) injection into the dorsum of the paw has been documented to elicit hyperalgesia 1 to 3 days after administration, lasting for 3 to 6 weeks (Fu et al., 2001).

To evaluate hyperalgesia and thermal sensitivity in rodents, the hot plate test was employed (Figure 2C; Supplementary Dataset S1). Formalin injection in the plantar region of the hind paw resulted in a significant reduction (*p* < 0.001) in withdrawal latency during thermal stimulation after 1 hour, with effects lasting until day four when compared to the control and E3330 groups. Furthermore, the formalin+E3330 group displayed significantly greater withdrawal latency (*p* < 0.001) compared to the formalin group, without significant differences when compared to the control and E3330 groups.

There were also non-significant increases in the contralateral paw’s response to thermal stimulation following formalin injection, indicating minimal effect on the opposite paw’s sensitivity (data not shown). Similar trends were observed in the von Frey test, where the formalin group exhibited a significantly lower paw withdrawal threshold (*p* < 0.05) in response to mechanical stimuli than the control and E3330 groups (Figure 2D; Supplementary Dataset S1). The formalin+E3330 group demonstrated a significantly higher (*p* < 0.05) paw withdrawal threshold one-hour post-E3330 administration compared to the formalin-treated animals, with this increase maintained until day four. Additionally, the E3330 groups exhibited no significant changes in basal withdrawal thresholds from day zero to day four, remaining comparable to the control group. Furthermore, administration of formalin in the contralateral hind paw did not affect the paw withdrawal threshold to mechanical stimuli (data not shown).

### 3.2 Formalin induces modulation in the expression profile of APE1/Ref-1

**Figure 3:**
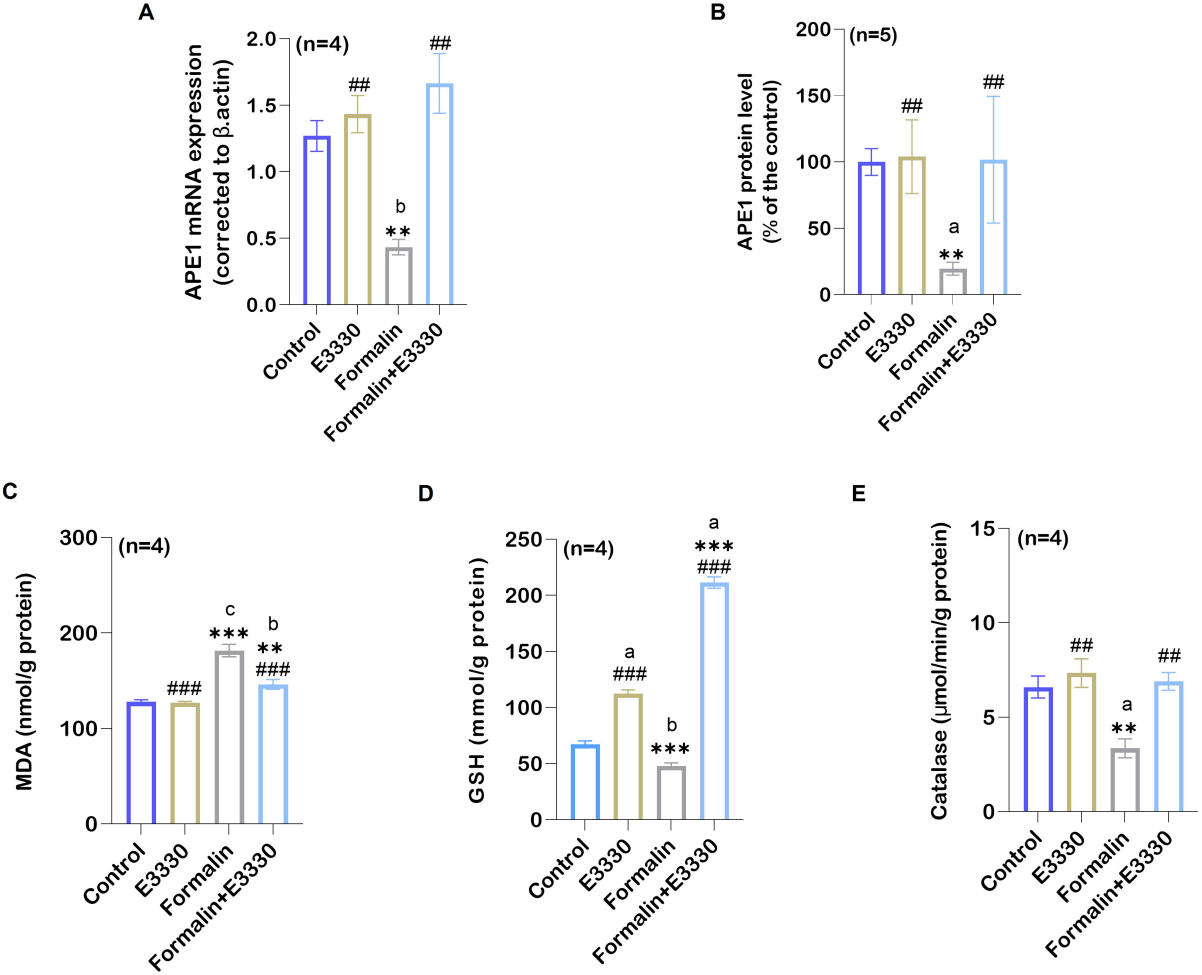
Formalin induces the reduction of the oxidative stress sensor APE1/Ref-1 in the spinal cord tissue and the consequence of its changes on the oxidative stress parameters of the examined groups. **(A)** A notable reduction was observed in the relative mRNA expression of APE1/Ref-1 compared to the control group (*n*=4 per group) as determined by qRT-PCR (^b^: *p* < 0.01 control vs. formalin). **(B)** Protein analysis in the ipsilateral spinal cord using ELISA showed an overall significant reduction after formalin injection in comparison to the control group (*n* = 5 per group). **(C)** Ipsilateral spinal cord tissue MDA levels in control, E3330, formalin and formalin+E3330 groups. The MDA content of the spinal cord is expressed as nmol/g of protein. A significant increase (*p* < 0.001) was observed in the formalin group compared to the control and E3330 groups. **(D)** Glutathione content in the spinal cord. Glutathione content is expressed as mmol/g of protein. A marked reduction (*p* < 0.01, *p* < 0.001) of the GSH level in the ipsilateral spinal cord tissue was observed in the formalin-injected group compared to the control and E3330 groups, respectively. On the other hand, the administration of E3330 reverses the formalin effect in the formalin+E3330 group. **(E)** Catalase levels in the ipsilateral spinal cord showed a significant reduction in the formalin-injected group (*p* < 0.001, *p* < 0.01) compared to the control and E3330 groups, respectively. Moreover, administration with E3330 returned the catalase level to its normal level. Data represent the mean ± SEM; ^a^ *p*<0.05, ^b^ *p*<0.01, ^c^ *p*<0.001 versus control group. ^*^ *p* < 0.05, ^**^ *p* < 0.01, ^**^ *p*<0.001 versus E3330 group. ^#^ *p*<0.05, ^##^ *p*<0.01, ^###^ *p*<0.001 versus formalin group.

The expression of oxidative stress sensor APE1/Ref-1 was detected in formalin-induced inflammatory conditions and after blocking APE1 redox function with E3330 in the ipsilateral spinal cord tissues (Figures 3A,B; Supplementary Dataset S1). APE1 expression revealed a significant reduction in the formalin-treated group as compared to the control in both mRNA amount and protein level (*p* < 0.01 and *p* < 0.05 respectively). The E3330 administration 1 hour before formalin induction and at day 3 post formalin induction significantly (*p* < 0.001 and *p* < 0.05) increased the mRNA gene expression and protein levels compared to the formalin group and normalized their levels to both the control and E3330 group.

### 3.3 E3330 ameliorates formalin-induced oxidative stress indices

The possible interplay between the blocking of APE1 redox function by E3330 and other redox systems following formalin induction was evaluated using oxidative stress-related biomarkers particularly MDA, GSH, and catalase (Figures 3C–E; Supplementary Dataset S1). Formalin injection caused a significant (*p* < 0.001) increase in the MDA level in the ipsilateral spinal cord tissue compared to the control group. E3330 administration 1 hour before formalin induction and at day 3 post formalin induction prevented the increase in MDA levels in the formalin+E3330 group maintaining control values (*p* < 0.05 vs. formalin group).

GSH level displayed a significantly (*p* < 0.01) decreased value in the ipsilateral spinal cord of formalin-injected rats as compared to control. Administration of E3330 1 hour prior to formalin induction and on the third day following formalin induction triggered a significant (*p* < 0.001) increase in GSH level in the formalin+E3330 group compared to other groups (Figure 3D). Furthermore, the E3330 group showed a significant (*p* < 0.05) increase in GSH level relative to the control group.

Catalase activity in ipsilateral spinal cord tissues was reduced significantly (*p* < 0.05) in the formalin group compared to control rats (Figure 3E). In contrast, E3330 injection 1 hour before formalin induction and at day 3 post formalin induction in the formalin+E3330 group significantly (*p* < 0.01) increased the catalase activity in the ipsilateral spinal cord tissues compared to the formalin group which assisted in inhibiting formalin-induced oxidative stress.

Free radical scavenging activity was determined according to the elimination of DPPH radicals, which is a validated method for screening the antioxidant activity (Bondet et al., 1997) (Table 3; Supplementary Dataset S1). Formalin induction resulted in a significant (*p* < 0.001) reduction in DPPH elimination after 3, 15, and 30 min (*p* < 0.01) as compared to control. Formalin induction also exhibited a significant (*p* < 0.001) reduction in DPPH elimination compared to the E3330 group after 3 min. Administration of the E3330 compound 1 hour prior to formalin induction and on day 3 following formalin induction displayed a significant (*p* < 0.01, *p* < 0.05) increase in the DPPH elimination percentage after 3 min as compared to the control group.

**Table 3:** DPPH radical scavenging percentage in the ipsilateral spinal cord tissue of the experimental groups.

Experimental groups	DPPH inhibition percentage (%) at different times		
	DPPH inhibition% after 3 min	DPPH inhibition% after 15 min	DPPH inhibition% after 30 min
Control	27.5 ± 0.5	65.37 ± 0.3	59.39 ± 1.5
E3330	32.16 ± 1.09 ^b/###^	71.84 ± 0.7 ^a/###^	68.28 ± 0.43 ^a/###^
Formalin	19.41 ± 0.4 ^c/***^	51.78 ± 2.5 ^c/***^	50 ± 1.22 ^b/***^
Formalin+E3330	32.35 ± 0.4 ^b/###^	64.72 ± 0.71 ^*/###^	67.64 ± 1.38 ^a/###^

Data represent the mean ± SEM (*n* = 3 per group). ^a^ *p* < 0.05, ^b^ *p* < 0.01, ^c^ *p* < 0.001 versus control group. * *p* < 0.05, ** *p* < 0.01, *** *p* < 0.001 versus E3330 group. ^#^ *p* < 0.05, ^##^ *p* < 0.01, ^###^ *p* < 0.001 versus formalin group.

### 3.4 E3330 improves formalin-induced alterations of mitochondrial morphology

**Figure 4:**
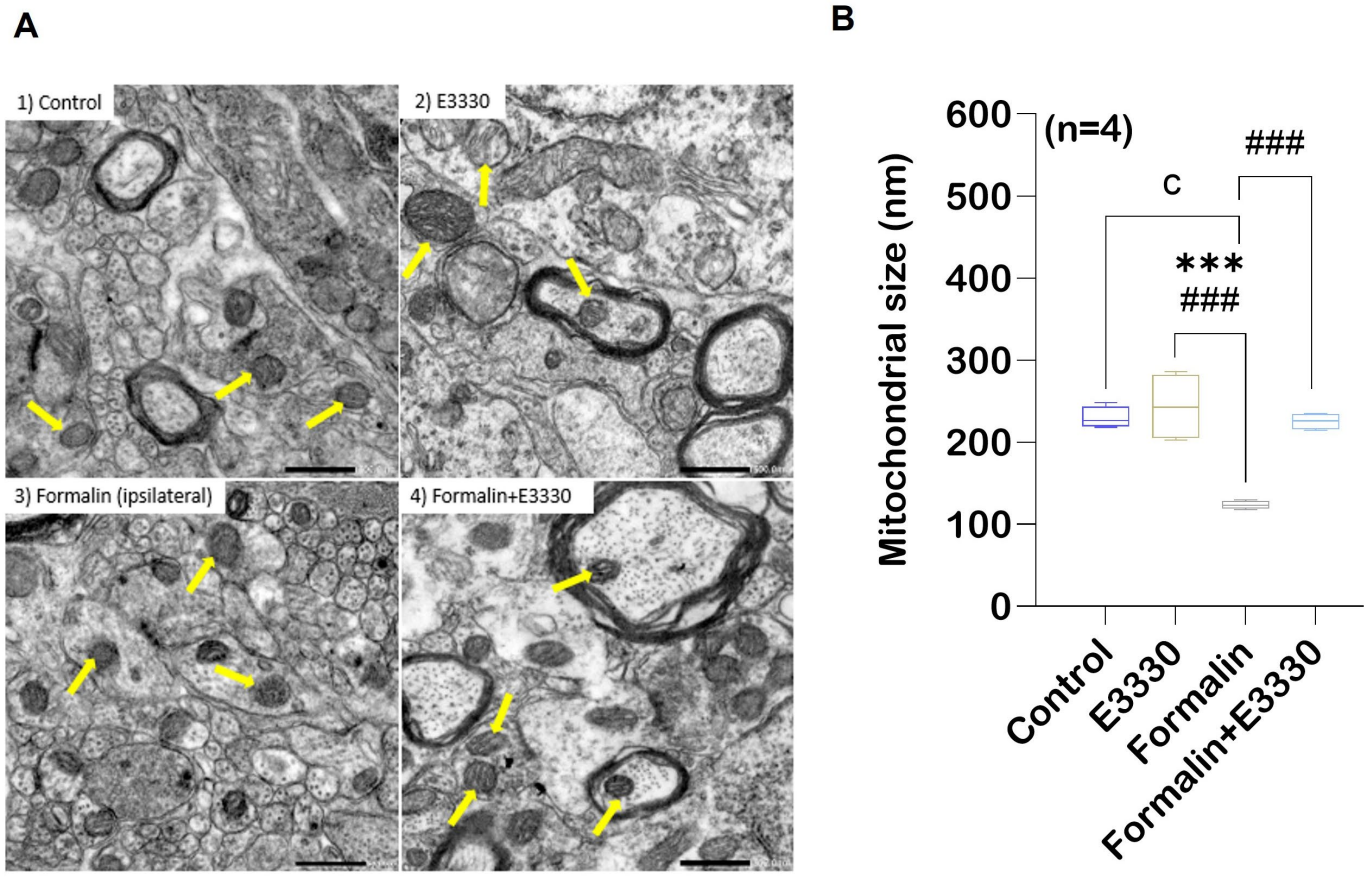
Formalin induces changes in the mitochondrial morphology in the spinal cord tissue. TEM showing variation in mitochondrial morphology and size in rat spinal cord gray matter. **(A)** Normal mitochondrial morphology in the control group. In the E3330 group, mitochondria similar to the control are found. formalin-induced ipsilateral sections showed mitochondrial degeneration with fracture and fission of cristae (yellow arrows) in the ipsilateral of the spinal cord. Mitochondria of the formalin+E3330 group retained a control-like morphology. **(B)** Quantitative analysis showed changes in mitochondrial diameter (nm) in the different experimental groups. Results are expressed as mean ± SEM (*n* = 4). ^a^ *p*<0.05, ^b^ *p*<0.01, ^c^ *p*<0.001 versus control group. ^*^ *p*<0.05, ^**^ *p*<0.01, ^***^ *p*<0.001 versus E3330 group. ^#^ *p*<0.05, ^##^ *p*<0.01, ^###^ *p*<0.001 versus formalin-induced group. Scale bar 500 nm.

Recently, the etiology of pain has been related to mitochondrial malfunction (Silva Santos Ribeiro et al., 2022). Overexpression of ROS can target mitochondria and trigger cell death (Andrés Juan et al., 2021). Therefore, we used transmission electron microscopy to examine changes in mitochondrial morphology in lamina II of the dorsal spinal cord, ipsilateral to the formalin injection, in the different experimental groups (Figure 4A; Supplementary Dataset S1).

Mitochondrial examination of the control rats revealed a normal appearance with an average size of 230 nm. Moreover, the control mitochondria showed well-defined organization with a two-membrane structure and distinct cristae, as outlined in (Figure 4B). The tissue mitochondria of the E3330 group displayed a distinctive structural organization, with an average size of 243.48 nm, well-defined cristae, and a central matrix. In contrast, formalin induction led to an increase in the thickness of the mitochondrial outer membrane, accompanied by disorganization within its compartments and a decrease in cristae density. In addition, significant (*p* < 0.001) decrease in the size of mitochondria with an average of 123.28 nm was observed in this formalin group compared to both the control and E3330 groups. On the other hand, microscopic analysis of the formalin+E3330 group revealed a healthy mitochondrial architecture, with an average size of 225.38 nm, similar to the control group. This observation suggests that the administration of E3330, 1 hour before formalin induction and on day 3 post formalin induction, effectively prevented morphological alterations, thus preserving the typical mitochondrial structure. Additionally, the integrity of the outer membrane and cristae was maintained.

### 3.5 APE1/Ref-1 influences inflammatory signaling cascades

**Figure 5:**
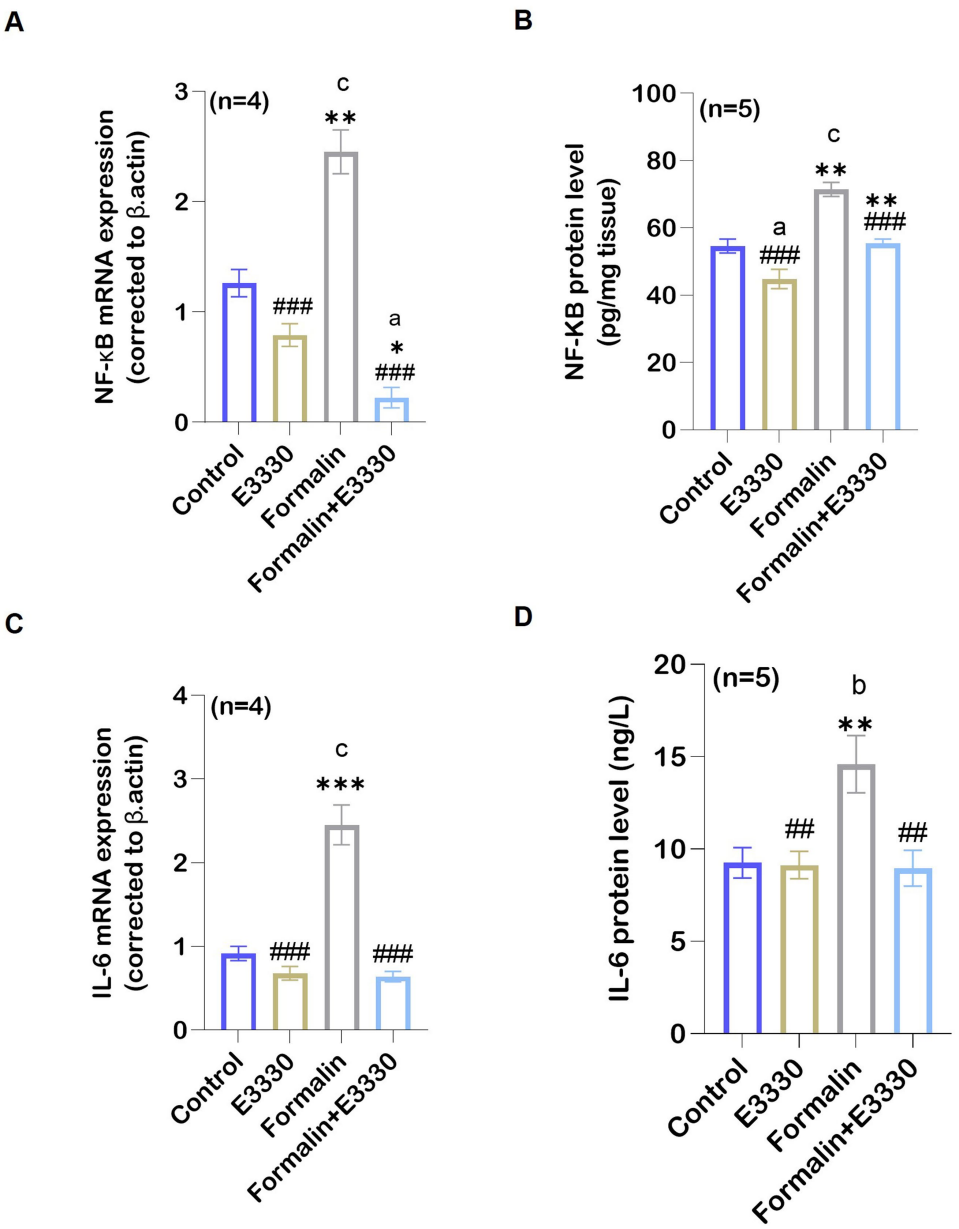
Formalin regulated the expression of APE1/Ref-1 targets: **(A)** Following formalin injection, qRT-PCR analysis of NF-κB mRNA in the spinal cord (*n*=4 per group) indicated a significant increase (^c^: *p* < 0.001 formalin vs control). This increase is reversed by E3330 administration (^a,*,###^: *p* < 0.001, formalin+E3330 vs control, E3330 and formalin group, respectively). **(B)** In addition, formalin injection induced a significant elevation in NF-κB protein level (*n* = 5 per group) (^c^: *p* < 0.001 formalin vs control; ^**^:*p* < 0.01 formalin vs E3330). In contrast, NF-κB activation upon E3330 administration remained limited (^###^: *p* < 0.001, formalin+E3330 vs formalin). **(C)** qRT-PCR analysis of the pro-inflammatory cytokine IL-6 in the spinal cord demonstrated a significant increase upon formalin induction (^c^: *p* < 0.001, formalin vs control and ^***^: *p* < 0.001 formalin vs E3330). **(D)** Protein analysis of IL-6 using ELISA demonstrated parallel results consistent with IL-6 mRNA expression (^b,**^: *p* < 0.01 formalin vs control and E3330 group; ^##^: *p* < 0.01, formalin+E3330 vs formalin). Relative expression values are calculated in reference to the control group. Data represents the mean ± SEM: ^a^ *p* < 0.05, ^b^ *p* < 0.01, ^c^ *p* < 0.001 versus control group. ^*^ *p* < 0.05, ^**^ *p* < 0.01, ^***^ *p* < 0.001 versus E3330 group. ^#^ *p* < 0.05, ^##^ *p* < 0.01, ^###^ *p* < 0.001 versus formalin group.

APE1 acts as a “hub-protein” that controls pathways relevant to inflammation by modulating the DNA-binding activity of the transcription factor NF-κB, a key player of immune and inflammatory signaling pathways (Thakur et al., 2014). The expression of NF-κB was recognized as a master switch that is essential for triggering and maintaining the immune responses. NF-κB induces the expression of various pro-inflammatory genes, including those encoding cytokines (Liu et al., 2017). Assessment of NF-κB mRNA expression using qRT-PCR and protein levels using ELISA provided consistent results, pointing to a significant (*p* < 0.001) increase in the ipsilateral spinal cord of the formalin group as compared to the control group (Figures 5A,B; Supplementary Dataset S1). In contrast, a significant (*p* < 0.001) reduction of NF-κB mRNA expression and protein levels was found in the formalin+E3330 group as compared to the formalin group, with levels similar to the control and E3330 groups. The E3330 group showed no significant changes in NF-κB mRNA expression and protein levels as compared to the control group.

IL-6 is a bioactive protein produced in response to tissue damage. It plays a role in inflammation and pain sensitization as reviewed by Sebba (2021). The IL-6 mRNA expression and protein levels revealed a significant increase in the formalin group as compared to the control and E3330 group (*p* < 0.001, *p* < 0.05, respectively) (Figures 5C,D; Supplementary Dataset S1). In contrast, E3330 injection 1 hour prior to formalin induction and on the third day following formalin induction caused a significant reduction in IL-6 mRNA expression and protein levels (*p* < 0.001 and *p* < 0.001, respectively) in the formalin+E3330 group as compared to the formalin group.

### 3.6 E3330 suppresses formalin-induced release of pro-inflammatory markers by modulating NLRP3 and caspase-1

**Figure 6:**
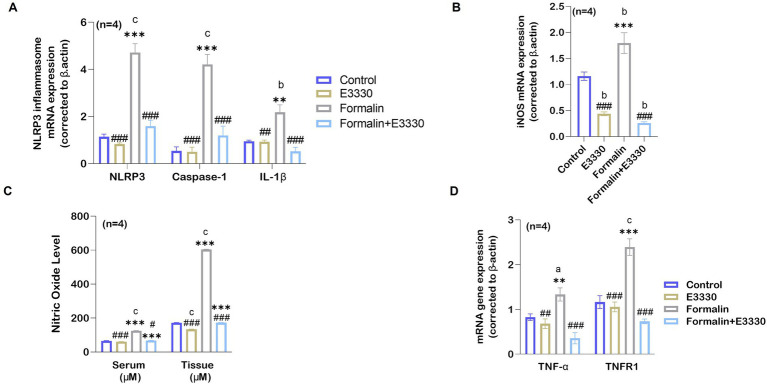
E3330 alleviates the release of pro-inflammatory mediators in formalin-induced inflammatory pain. **(A)** qRT-PCR analysis of the NLRP3 inflammasome mRNA relative expression and its upregulated genes Casapase-1 and IL-1β (*n* = 4 per group) in the spinal cord showed a significant increase (^c^: *p* < 0.001) in following formalin induction as compared to control rats. This increase goes back to normal level upon E3330 administration. **(B)** mRNA relative expression of iNOS shows an increase in the formalin group compared to other groups (^b^: *p* < 0.01 formalin vs control; ^***^: *p* < 0.001, formalin vs E3330). **(C)** Nitric oxide levels in the serum and the spinal cord displayed a significant increase after formalin injection (*n* = 4 per group; ^c^: *p* < 0.001, formalin vs control). Administration of formalin with E3330 inhibits the formalin-induced increase (^###^: *p* < 0.001, formalin+E3330 vs formalin). **(D)** Following formalin injection, qRT-PCR analysis shows a significant elevation of TNF-α mRNA (*n* = 4, ^a^: *p* < 0.05, formalin vs control; ^**^: *p* < 0.01, formalin vs E3330) and TNFR1 mRNA (*n* = 4, ^a^: *p* < 0.05, formalin vs control; ^*^: *p* < 0.05 E3330 vs formalin) expression. On the contrary, E3330 administration limits the increased expression of TNF-α mRNA (^##^: *p* < 0.01, E3330 vs formalin; ^###^: *p* < 0.001, formalin+E3330 vs formalin) and TNFR1 mRNA (^###^: *p* < 0.05, E3330 vs formalin; ^###^: *p* < 0.01 formalin+E3330 vs formalin). Relative expression values are calculated in reference to the control group. Data represents the mean ± SEM: ^a^ *p* < 0.05, ^b^ *p* < 0.01, ^c^ *p* < 0.001 versus control group. ^*^ *p* < 0.05, ^**^ *p* < 0.01, ^***^ *p* < 0.001 versus E3330 group. ^#^ *p* < 0.05, ^##^ *p* < 0.01, ^###^ *p* < 0.001 versus formalin group.

Our results showed a significant increase (*p* < 0.001) in the mRNA level of NLRP3 following formalin induction compared to the control and E3330 groups. Interestingly, the administration of E3330 mitigated this increase to normalization (Figure 6A; Supplementary Dataset S1). In addition, we measured the level of caspase-1 which is activated via proximity-induced autocatalytic activation upon recruitment to an inflammasome (Kelley et al., 2019). The results are in line with NLRP3 profile (Figure 6A; Supplementary Dataset S1).

On the other hand, it has been found that dysfunctional mitochondria can enhance NLRP3 activation, contributing to increased IL-1β production and exacerbating pain responses (Silva Santos Ribeiro et al., 2022). The cytokine interleukin 1β (IL-1β) has been implicated in pain and inflammatory processes at multiple levels, both peripherally and centrally (Ren and Torres, 2009). IL-1β serves as a pivotal mediator of the inflammatory cascade. Integral to the host-defense mechanisms against pathogens, it also plays a role by intensifying injury progression in chronic diseases and acute tissue damage (Lopez-Alvarez et al., 2018). Consequently, the analysis of IL-1β expression using qRT-PCR revealed a significant increase (*p* < 0.01) in the formalin-induced rats versus the control group (Figure 6A; Supplementary Dataset S1). Besides, the administration of E3330 did not cause any significant difference in the level of IL-1β mRNA relative expression in the formalin+E3330 group compared to the control and E3330 groups. As expected, the expression of IL-1β cytokines displayed no significant differences in the E3330 group versus the control group.

Increased production of the gaseous mediator NO is correlated with the release of cytokines involved in inflammatory processes (Wojdasiewicz et al., 2014). To further examine the consequences of IL-1β activation, we determined the mRNA expression of the iNOS enzyme, which controls NO production, as a relevant parameter to assess formalin-induced inflammation in rats (Figure 6B; Supplementary Dataset S1). The expression of iNOS mRNA in the ipsilateral spinal cord showed a significant increase (*p* < 0.01) in the formalin group versus the control group. In contrast, a significant (*p* < 0.001) reduction in the level of iNOS mRNA relative expression was observed in the formalin+E3330 group as compared to the control group. Additionally, the E3330 group showed a significant (*p* < 0.01) reduction in the level of iNOS mRNA versus the control group.

In addition, we measured NO levels in both serum and spinal cord tissues (Figure 6C; Supplementary Dataset S1). The results indicated a significant increase (*p* < 0.001) in the levels of NO in both the serum and ipsilateral spinal cord tissue in the formalin group as compared to the control group. In contrast, E3330 administration 1 hour before formalin induction and at day 3 post formalin induction provoked a significant (*p* < 0.001) decrease in NO level in both the serum and spinal cord in the formalin+E3330 group as compared to the formalin group. On the other hand, the E3330 group displayed a significant (*p* < 0.05) decrease in the NO serum level as compared to the control group. On the contrary, the E3330 group showed no differences in the level of spinal NO when compared to the control group.

Tumor necrosis factor-alpha (TNF-*α*), is a key pro-inflammatory cytokine that drives cytokine storms and stimulates a cascade of other cytokines in pain-related pathways (Duan et al., 2022). Therefore, we determined the mRNA expression of TNF-α and its receptor TNFR1 (Figure 6D; Supplementary Dataset S1). Analysis of TNF-α mRNA expression displayed a significant (*p* < 0.05) increase in formalin-induced rats versus control rats whereas, administration of E3330 1 hour prior to formalin induction and on the third day following formalin induction in the formalin+E3330 group reduced this elevation and kept the level of TNF-*α* similar to that of the control group. On other hand, TNFR1 mRNA significantly (*p* < 0.05) increased in the formalin group compared to the control group. The E3330 group displayed a significant (*p* < 0.01) reduction in the level of TNFR1 when compared to the formalin group. The E3330 group also showed no changes in the level of TNFR1 when compared to the control group. This indicates that E3330 has a therapeutic effect in inflammatory pain conditions by reducing the inflammation caused by elevation of TNF-α and TNFR1.

### 3.7 E3330-mediated upregulation of the IL-10 anti-inflammatory cytokines and downregulation of the BDNF–TrkB and GDNF neurotrophic factors following formalin induction

**Figure 7:**
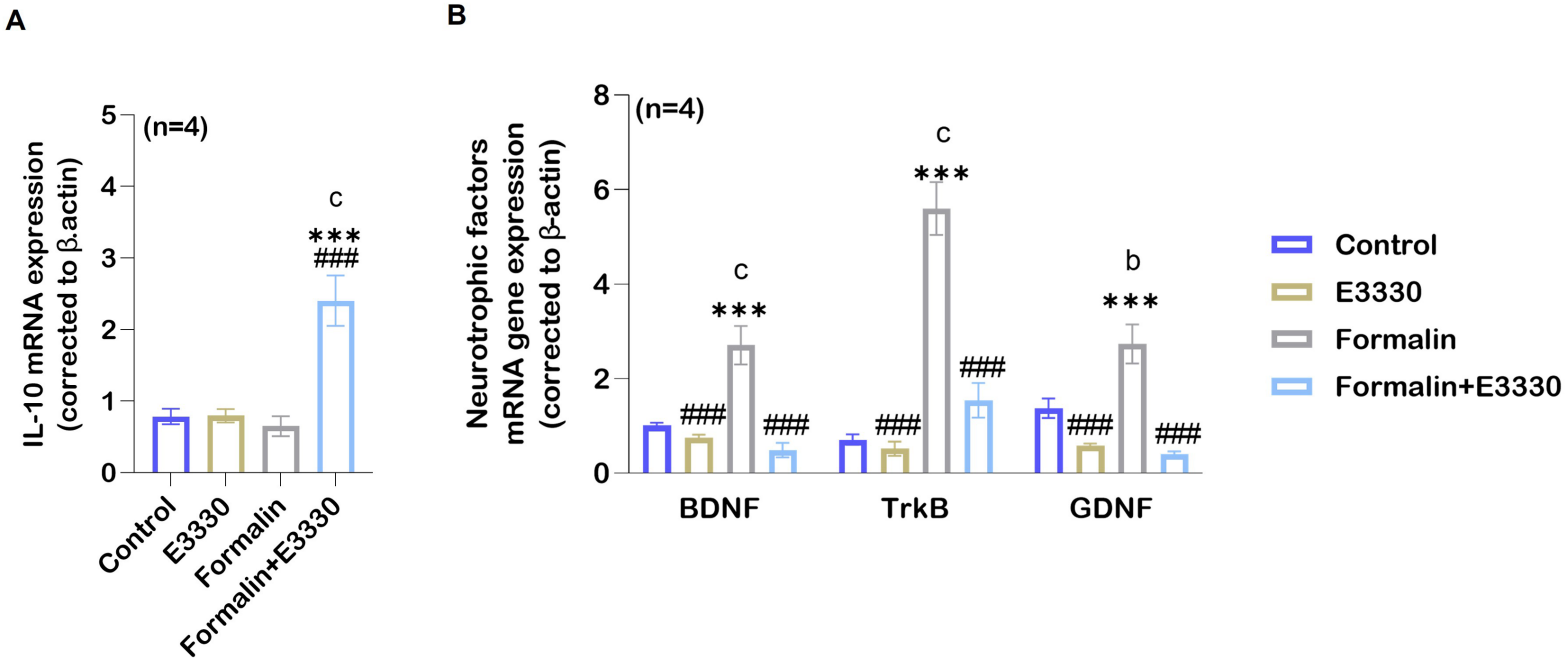
Formalin downregulates the expression of anti-inflammatory cytokines IL-10 following inflammatory pain induction. **(A)** Anti-inflammatory cytokine IL-10 mRNA expression levels increase in the spinal cord in the formalin+E3330 group as compared to other groups (^c^: *p* < 0.001, formalin+E3330 vs control; ^***^: *p* < 0.001, formalin+E3330 vs E3330; ^###^: *p* < 0.001, formalin+E3330 vs formalin). In contrast, no changes were detected in the other groups. **(B)** qRT-PCR analysis of BDNF, TrkB and GDNF mRNA in the spinal cord indicated a significant increase after formalin injection (*n*=4; ^c^: *p* < 0.001, ^b^: *p* < 0.01, formalin vs control, respectively). In contrast, E3330 administration induced a significant decrease in neurotrophins expression (BDNF and its receptor TrkB besides GDNF): ^###^: *p* < 0.001, formalin+E3330 vs formalin. Data represents the mean ± SEM: ^a^ *p* < 0.05, ^b^ *p* < 0.01, ^c^ *p* < 0.001 versus the control group. ^*^ *p* < 0.05, ^**^ *p* < 0.01, ^***^ *p* < 0.001 versus E3330 group. ^#^ *p* < 0.05, ^##^ *p* < 0.01, ^###^ *p* < 0.001 versus the formalin group.

Interleukin-10 (IL-10) is an effective anti-inflammatory cytokine that pleiotropically inhibits the activity of numerous pro-inflammatory factors (Vanderwall and Milligan, 2019). To check the antioxidant activity of E3330 during formalin-induced inflammatory pain conditions, we determined IL-10 mRNA expression in the ipsilateral spinal cord (Figure 7A; Supplementary Dataset S1). Analysis of the IL-10 mRNA relative expression showed a significant (*p* < 0.001) increase in the (formalin+E3330) group as compared to the control and formalin groups. In contrast, the formalin group showed no significant changes (*p* = 0.96) in the level of IL-10 when compared to the control group. In addition, the administration of E3330 resulted in non-significant changes in the expression of IL-10 mRNA as compared to the control group.

More recently, BDNF and GDNF are now recognized as key pain modulators, as their receptors are found on nociceptors and contribute to pain sensitization and chronic pain development (Ferrini et al., 2020). Accordingly, we determined whether the expression of neurotropic factors such as BDNF and its receptor TrkB was influenced by formalin-induced inflammatory pain. Microglia is the major source for the synthesis and release of BDNF, which is relevant for increasing neuronal excitability by causing disinhibition in dorsal horn neurons of the spinal cord (Coull et al., 2005). Moreover, primary afferent-derived BDNF has a pro-nociceptive role in mediating the transition from acute to chronic pain (Sikandar et al., 2018). GDNF has been shown to activate transient receptor potential vanilloid 1 (TRPV1) responses, leading to behavioral sensitivity to heat and cold (Elitt et al., 2006). In addition, the GDNF/GFRα1 signaling pathway interferes with inflammatory pain, through the activation and sensitization of non-peptidergic neurons as reviewed by Morel et al. (2020). Therefore, we investigated the effect of blocking APE-1 redox activity by E3330 on BDNF–TrkB and GDNF mRNA levels. Consequently, the relative BDNF–TrkB and GDNF mRNA expression was significantly increased (*p* < 0.001, *p* < 0.01, respectively) in the formalin group as compared to the control group (Figure 7B; Supplementary Dataset S1). The application of E3330 1 hour before and day 3 post the formalin induction in the formalin+E3330 group prevented neurotrophic factor changes and maintained a basal level of BDNF and GDNF mRNA, similar to the control group conditions. On the other hand, the formalin+E3330 group triggered a significant decrease (*p* < 0.001) in both BDNF–TrkB and GDNF expressions as compared to the formalin group condition. Rats that received E3330 only displayed no significant changes (*p* = 0.84 and *p* = 0.13) in the level of BDNF and GDNF as compared to the control group whereas, a significant decrease (*p* < 0.001) was found relative to the formalin group.

### 3.8 APE1/Ref-1 redox activity blockade modulated dopamine release in formalin-induced inflammatory pain condition

**Figure 8:**
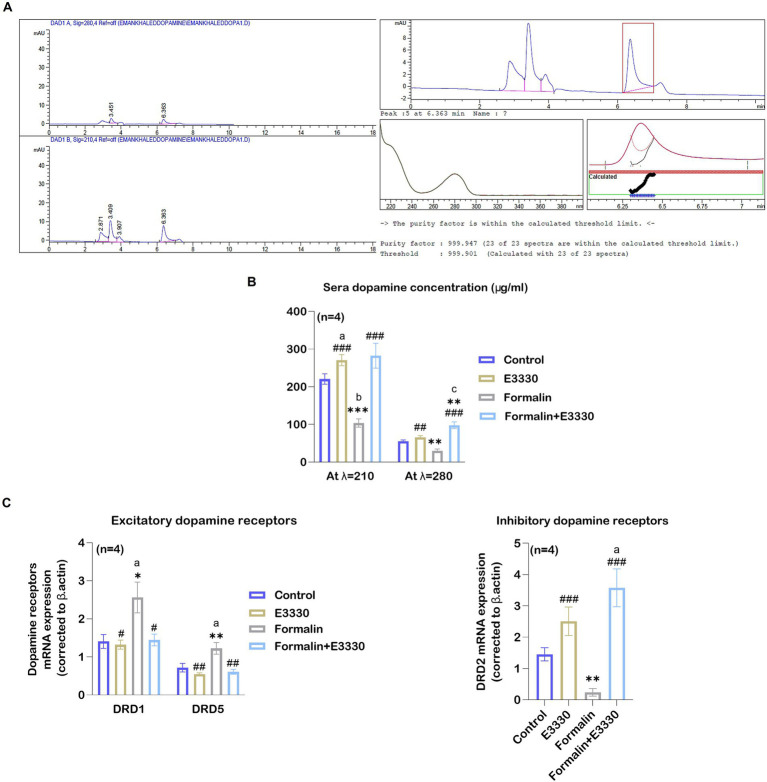
E3330 induced alterations of DA and DA receptors expression. **(A)** Chromatograms of the DA standard revealed peak areas of 35.48779 [mAU^*^s] at 280 nm and 121.92159 [mAU^*^s] at 210 nm, alongside spectral and purity factor details for the DA standard. **(B)** The HPLC quantitative analysis of DA concentration at 280 and 210 nm wavelengths displayed a significant reduction after formalin injection (b: p<0.01, formalin vs control; ^***^ *p* < 0.001, ^**^ *p* < 0.01, formalin vs E3330; respectively). On the contrary, E3330 administration induced a significant increase (^###^: p<0.001, formalin+E3330 vs formalin). **(C)** Changes in DRD1, DRD5, and DRD2 mRNA expression level in the spinal cord; qRT-PCR analysis indicated a significant increase in DRD1 and DRD5 mRNA expression after formalin injection (^a^: *p* < 0.05, formalin vs control; ^*^ *p* < 0.001, formalin vs E3330 and ^**^ *p* < 0.01, formalin vs E3330, respectively), while it showed a significant reduction in D2R mRNA expression after formalin injection (^**^: *p* < 0.01, formalin vs E3330). Moreover, E3330 administration induced a significant increase in DRD2 mRNA expression (^a^: *p* < 0.05, formalin vs control; ^###^: *p* < 0.001, formalin+E3330 vs formalin). In contrast, E3330 administration resulted in decreased DRD1 and DRD2 mRNA level (^#^: *p* < 0.05, formalin+E3330 vs formalin; ^##^: *p* < 0.01 formalin+E3330 vs formalin, respectively). Data are expressed as mean ± SEM (*n* = 4). a *p* < 0.05, b *p* < 0.01, c *p* < 0.001 versus control group. ^*^ p<0.05, ^**^ *p* < 0.01, ^***^ *p* < 0.001 versus E3330. ^#^ *p* < 0.05, ^##^ *p* < 0.01, ^###^ *p* < 0.001 versus the Formalin group.

DA can function either as an excitatory mechanism or as an inhibitory mechanism in the central nervous system depending on the location of dopamine neurons and the receiving characteristics of the next neuron in the chain (Klein et al., 2019). Besides, DA contributes to the progression of subacute to chronic pain (Serafini et al., 2020). DA dysfunction, as a consequence of the oxidative stress involved in health and disease, induces minor injuries that can heighten the experience of pain (Juárez Olguín et al., 2016). Therefore, we tested the effect of E3330 on the regulation of DA release. The standard DA peak was eluted at a retention time of 6.363 min (Figure 8A) at wavelength 210 nm. The results of DA concentration in the sera samples were summarized in Figure 8B and Supplementary Dataset S1. Our results at 210 nm wavelength indicated a significant decrease (*p* < 0.01) in the formalin group as compared to the control group (Figure 8B). Whereas the formalin group displayed a significant decrease (*p* < 0.001) compared to the E3330 group. The results were confirmed using a spectrum at 280 nm, wavelength which showed a compatible significant decrease (*p* < 0.01) in the formalin group versus the E3330 group (Figure 8B). On the other hand, administration of E3330 1 hour before formalin induction at day 3 post formalin induction in formalin+E3330 group caused a significant increase (*p*0.001>) in the DA concentration compared to the control at 280 nm wavelength with no significant change at 210 nm wavelength. Maybe this is due to the huge interferences from other catecholamine in the sample. In addition, inhibition of the redox activity of APE1 in inflammatory pain conditions by E3330 in the formalin+E3330 group displayed a significant increase in the DA concentration at the 280 nm wavelength as compared to the formalin and E3330 groups (*p*0.001 > and p0.01>, respectively). The present data also showed a significant (*p* > 0.01 and *p* > 0.001) increase in the DA concentration at 280 and 210 nm wavelength, respectively in the E3330 group compared to the formalin group, while no changes were found relative to the control group.

### 3.9 Formalin and E3330 differentially alter dopamine signaling

Previous studies have demonstrated the importance of the hypothalamic-spinal DAergic system in controlling pain and suggested that D1 and D2-like receptors play a role in DA-mediated anti-nociceptive and pro-nociceptive actions (Puopolo, 2019). For this purpose, we tested the effect of blocking the redox function of APE1 using E3330 on the DA receptor subtypes, especially D1, D5, and D2 receptors. Accordingly, we detected the relative expression of DRD1, DRD5, and DRD2 mRNA in the ipsilateral spinal cord of the different experimental groups (Figure 8C; Supplementary Dataset S1). Our results revealed a significant (*p* > 0.05, *p* > 0.01) elevation in the DRD1 and DRD5 mRNA expression following formalin induction in the formalin group as compared to the control and E3330, respectively. The administration of E3330 in the formalin+E3330 group displayed a significant (*p* > 0.05, *p* < 0.01) decrease in DRD1 and DRD5 mRNA expression, respectively when compared to the formalin group. Moreover, the DRD1 and DRD5 mRNA levels in the formalin+E3330 group remained like that of the control group with non-significant change. On the contrary, the level of DRD2 mRNA showed a significant (*p* < 0.01) decrease in the formalin group compared to the E3330 group and displayed a significant increase in the formalin+E3330 group when compared to the control and formalin groups (*p* > 0.05 and *p* > 0.001, respectively). Moreover, the E3330 group showed a significant increase (*p* < 0.01) in DRD2 mRNA expression as compared to the formalin group.

### 3.10 Molecular docking of E3330 on DA receptors

**Figure 9:**
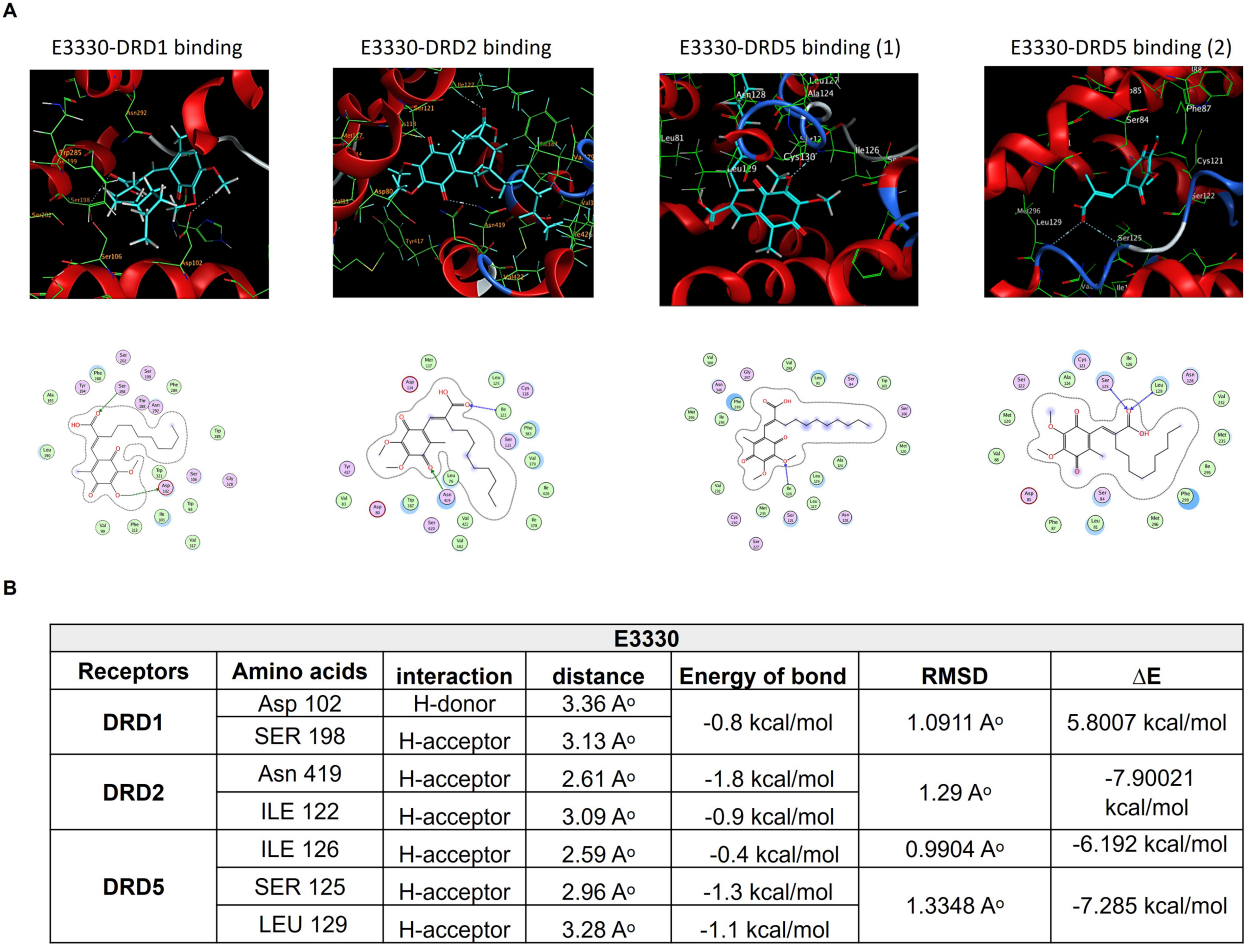
Mode of interactions of the APE1/Ref-1 inhibitor E3330 compound (E3330-depicted in cyan sticks) with the active site of DA receptors DRD1, DRD2, and DRD5. **(A)** The binding interactions are represented through an H-bond (gray dot sticks), as shown in the 3D model. Besides, the corresponding amino acid residues' binding modes for each receptor and the DA structure were represented in the 2D model as well. **(B)** The docked poses of the E3330 compound in the binding site of DRD1, DRD2, and DRD5 DA receptors are represented through docking score parameters such as distance, binding free energy, and interaction between the compound and the key residues for the receptors' active site binding. The best-docked pose was selected based on RMSD, which must be less than 2Aᵒ, and the overall pose binding energy (∆E) represents the binding affinity of the compound. These results are the mean of 3 runs. These figures were generated using the Molecular Operating Environment (MOE 2014.13) software.

**Figure 10:**
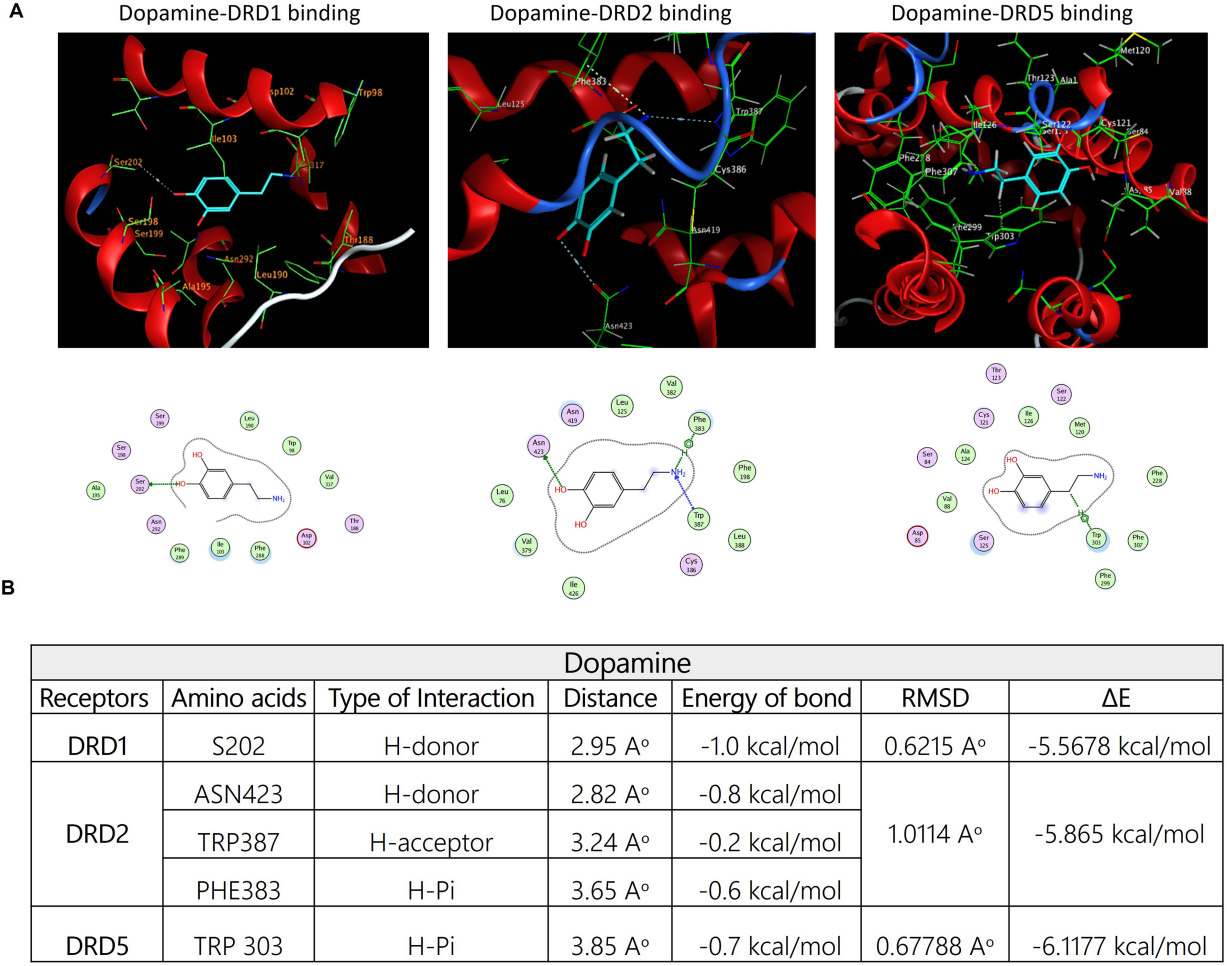
**(A)** Three-dimensional binding modes and interactions of dopamine (DA-depicted in cyan sticks) superimposed on the active sites of DRD1, DRD2, and DRD5 receptors, respectively (depicted in green sticks). The potential binding interactions between dopamine and amino acid residues on the active site of each receptor were symbolized using gray dotted sticks. the corresponding amino acid residues' binding modes for each receptor and the DA structure were represented in the 2D model as well. **(B)** The possible binding between DA and the three receptors is shown in the table, which exhibits docking score parameters such as the distance, binding free energy, and interaction between the compound and the key residues for the receptor active site binding. The best-docked pose was selected based on RMSD, which has to be less than 2Aᵒ and the overall pose binding energy (∆E) represents the binding affinity of the compound. These results are the mean of 3 runs and the figures were generated using Molecular Operating Environment (MOE 2014.13) software.

Consistent with our earlier findings and given the critical function of the dopaminergic system in pain regulation, there is an increasing interest in the utilization of multimodal analgesic regimens that target distinct dopaminergic receptors; however, the complete illustration is yet lacking. Accordingly, we aimed to estimate the potential interaction of E3330 with the D1, D5, and D2 receptor subtypes of DA receptors, so we carried out an *in-silico* analysis as a prediction tool. Our molecular docking analysis demonstrated the possible interaction between E3330 with the excitatory DA receptors (D1 and D5) that are involved in inflammatory processes and the inhibitory dopamine receptor (D2) involved in anti-inflammatory processes (Figure 9). The predicted 3D structure is validated by comparison of the predicted binding site and the relative binding affinities of DA as a reference (Figure 10). Optimization and energy minimization processes of DA receptors and E3330 were performed for better geometry followed by active site detection. The data was validated by docking DA molecules with the receptors as a reference. The best model was selected based on root mean square scores (RMSD) and free binding energy. The best binding site pocket for DRD1 and DRD2 was selected according to the study of Kalani et al. (2004) and Zhuang et al. Zhuang et al. (2021). Since there is a shortage of information about the DRD5 binding pocket, we selected the same position as other receptors. The analysis displayed that the best position for DA binding was S202 residue in DRD1, Asn423, TRP387, and PHE383 in DRD2 and TRP303 residue in DRD5 (Figure 10A). The criteria for binding with acceptable RMSD are explained in Figure 10B. Additionally, we checked the best binding position of E3330 on DRD1, DRD2, and DRD5. We used DA-binding as a reference to determine the active site of the receptors. The best binding of E3330 on DA receptors was with Asp102 and SER198 residues in DRD1, Asn419, ILE122 residues in DRD2, and ILE126, SER125, and LEU129 residues in DRD5 (Figure 9A). The criteria of binding were explained in Figure 9B.

## 4 Discussion

Pain sensitization is a multifaceted phenomenon influenced by various mechanisms, with oxidative stress playing a critical role in inflammatory pain pathways that extend from peripheral nociceptors to central sensitization (Ferger et al., 2010; Chimenti et al., 2018). In this study, we employed subcutaneous formalin injection into the hind paw as a classical method to induce inflammatory and tonic pain, alongside long-term secondary hyperalgesia (Saito et al., 2005; Tulpule and Dringen, 2013).

The compound E3330 demonstrated significant efficacy by inhibiting paw volume increase, elevating pain thresholds, and alleviating pain-related behaviors, thereby suggesting its potential to alleviate both edema and nociception.

Several key cell types are implicated in the molecular changes modulated by E3330 (Kelley et al., 2011). Sensory neurons, which are pivotal in transmitting pain signals, may have their excitability altered by E3330, leading to reduced responsiveness to inflammatory mediators and thereby elevating pain thresholds. Astrocytes and microglia, which are activated in response to injury and inflammation, could also be influenced by E3330. Our findings suggest that E3330 may decrease microglial activation, resulting in a reduced release of pro-inflammatory cytokines that contribute to pain sensitization. Similarly, E3330 may modulate astrocytic activity, enhancing the resolution of inflammation. Additionally, E3330 appears to influence the activity of endothelial cells, contributing to a reduction in edema associated with inflammation. Various immune cells, including macrophages and mast cells, may be influenced by E3330, reducing the production of pro-inflammatory cytokines and thus affecting overall inflammatory responses and pain signaling.

A pivotal component of our investigation was APE-1/Ref-1, a multifunctional protein that regulates cellular functions and oxidative stress and has emerged as a key player in inflammatory states (Oliveira et al., 2022). APE/Ref-1 redox activity plays a critical role in regulating various gene and protein functions by enhancing the transcriptional activity of key transcription factors such as AP-1, NF-κB, p53, HIF-1α, Nrf2, Egr-1, and ATF3, while also modulating the expression of proteins involved in cell survival (e.g., survivin, Bcl-2), apoptosis (e.g., Bax, caspase-1), cell cycle progression (e.g., p21, cyclin D1), angiogenesis (e.g., VEGF), and inflammatory responses (e.g., iNOS, COX-2, NLRP3), thereby influencing processes like cell proliferation, differentiation, and stress response (Oliveira et al., 2022; Gampala et al., 2024).

Our observations showed that E3330, when administered with formalin induction, inhibited APE1 redox activity and stabilized its expression at both mRNA and protein levels. This finding is consistent with prior research by Zaky et al. (2018), which reported diminished APE1 expression in inflammatory pain models concomitant with significant improvement in the expression profile upon E3330 administration. Moreover, it corroborates the conclusions of Chu et al. (2014), which indicate that reduced APE1 expression under oxidative stress increases neuronal susceptibility to continuous oxidative stress challenges. This underscores APE1’s integral role in regulating inflammatory responses and oxidative stress in pain models (Sahakian et al., 2023). Furthermore, APE1 redox activity is critical as a coactivator of NF-κB, a transcription factor that promotes the expression of pro-inflammatory cytokines (Oliveira et al., 2022). Consistent with previous studies (Herzberg et al., 2019; Duan et al., 2022; Serafini et al., 2020) indices of oxidative stress such as malondialdehyde (MDA) levels, glutathione (GSH), catalase, and free radical scavenging activities were altered by E3330, which reversed the deleterious effects of oxidative imbalance. Ultrastructural analysis of spinal cord tissue revealed significant mitochondrial damage due to formalin induction; notably, pre-injection with E3330 had a favorable impact on mitochondrial viability, underscoring its therapeutic potential.

The increase in NF-κB expression observed post-formalin administration was counteracted by the pre-injection of E3330, leading to a notable reduction in both NF-κB gene expression and protein levels. Our findings corroborate those of Jedinak et al. (2011), which indicate that E3330’s effects are partially mediated by the inhibition of NF-κB. This evidence robustly supports E3330’s role in modulating NF-κB expression in response to inflammatory stimuli, highlighting its therapeutic potential in mitigating inflammatory pain via this pathway. As a result, formalin-induced inflammation led to mitochondrial dysfunction, characterized by structural abnormalities observed in electron microscopy, which correlated with NLRP3 inflammasome activation and subsequent Caspase-1-dependent cytokine release. Notably, E3330 administration mitigated these effects, preserving mitochondrial integrity and suppressing NLRP3 activation, thereby reducing the inflammatory cascade (Tang et al., 2021). Additional investigation could explore the interactions between E3330 and the cytochrome c (cyt-c)/NF-κB/inflammasome complex to deepen our understanding of these mechanisms.

Following formalin induction, we observed a significant increase in both mRNA and protein levels of IL-6, highlighting its upregulation in response to formalin-induced inflammatory conditions. This aligns with the findings by Sebba (2021), who emphasized IL-6’s crucial role in inflammatory pain. Other inflammatory mediators such as NO, iNOS, and IL-1β also exhibited stimulated expression following formalin induction. E3330 effectively suppressed iNOS and IL-1β expression, reinforcing its anti-inflammatory properties. While formalin-induced elevations in IL-1β mRNA expression were noted, these were inhibited by E3330, further supporting its role in modulating inflammatory pain (Sebba, 2021). Additionally, the data underscored those glial cells in the spinal cord produced TNF-*α* following formalin induction, which sensitizes nociceptive neurons. Our results indicated that formalin induction significantly raised TNF-α levels, consistent with findings by Zhang et al. (2011), which demonstrated TNF-α’s role in perpetuating inflammatory pain via TNFR1 activation of NMDA receptors. Gonçalves et al. (2020) further elaborated on TNF-α’s contribution to the “immune-to-brain” communication pathways that facilitate pain sensitization. Notably, E3330 administration significantly reduced levels of both TNF-α and TNFR1 (Gonçalves et al., 2020).

BDNF–TrkB and GDNF are critical factors in pathological pain condition reported to modulate nociceptive neurotransmission in the superficial dorsal horn of the spinal cord (Ferrini et al., 2020). Our investigation revealed that formalin exposure led to increased BDNF–TrkB and GDNF expressions., Both key modulators of pain sensitization. This increase may highlight how BDNF–TrkB signaling may involving in pain amplification through increased sensitivity of target sensory neurons (Ferrini et al., 2020). While GDNF is generally known for its neuroprotective and anti-nociceptive effects, its increased expression in formalin-induced inflammatory pain could be due to compensatory response. Our result was compatible with a recent review by Azevedo et al. (2020) which conclude that neuroinflammation induces GDNF expression in activated astrocytes and microglia, infiltrating macrophages, nestin-positive reactive astrocytes, and neurons/glia (NG2)-positive microglia-like cells. Interestingly, E3330 effectively abolished this up-regulation, by restoring restored BDNF–TrkB levels to baseline and decreasing GDNF expressions. Given its redox-regulating properties, E3330 likely exerts its effects by modulating inflammatory pathways, thereby attenuating BDNF-GDNF signaling. This regulatory influence suggests that E3330 may mitigate pain sensitivity and disrupt neuroimmune interactions that contribute to chronic pain progression. Further studies are still needed to better explain the possible interplay between E3330 and GDNF/GFRα1/Ret pathway.

In conclusion, E3330 exhibited a comprehensive analgesic effect by enhancing pain sensitization, modulating oxidative stress, inhibiting inflammatory cascades, and influencing neuro-inflammatory markers. Our findings advocate for E3330 as a promising novel analgesic strategy for chronic inflammatory pain, warranted by a deeper exploration of its underlying molecular mechanisms.

We also investigated E3330’s influence on pain signaling through dopamine neurotransmitter levels and receptor expression, given that dopamine regulates pain perception through descending pathways to the spinal cord (Lopez-Alvarez et al., 2018). Painful stimuli disrupt dopamine homeostasis, thereby contributing to chronic pain (Li et al., 2019). Our results indicated that formalin induction diminished serum dopamine levels, while E3330 restored these levels, reduced pain latency, and normalized the mRNA expression of DRD1 and DRD5, along with restoring DRD2 expression in the spinal cords of formalin-treated rats. This aligns with earlier observations regarding spinal D1 and D5 receptors and their roles in nociceptive hypersensitivity (De la Luz-Cuellar et al., 2023).

There remains a complexity in understanding why E3330 significantly reverses many formalin-induced behavioral and molecular changes, yet when administered alone, only affects some of these molecules. Further investigations could elucidate the nuances of these interactions.

Furthermore, to preliminary screen the potential of E3330 compound to interact with dopamine receptors, as a possible hypothesis that explain our biochemical finding of its effects on dopaminergic signaling, we employed bioinformatics analysis to get an overview on that.

Through in-silico modeling, we assessed potential direct interactions between E3330 and various dopamine receptors (DRD1, DRD2, and DRD5) using MOE software (MOE 2014.13). Our results supported our hypothesis that E3330 interacts with key active sites within the dopamine receptor pockets, resembling the binding behavior of dopamine itself (Martel and Gatti McArthur, 2020). This data provides valuable insights into the molecular mechanisms governing dopamine receptor functions (Zhuang et al., 2021).

Our study demonstrates that E3330 mitigates inflammatory pain by modulating dopaminergic signaling related to DRD1, DRD2, and DRD5 receptors while also impacting microglial activation. The formalin model elucidated E3330’s influence on both acute and chronic pain mechanisms, positioning it as a potential candidate for pain management and neuroimmune interventions. The urgency for novel therapeutic approaches is underscored, with APE1/Ref-1 emerging as a promising target for future research.

E3330 is currently under investigation for its possible involvement in pain sensitization mechanism through inhibition of APE1redox activity. By targeting APE1/Ref-1 mediated regulation of oxidative stress and inflammatory processes (Fehrenbacher et al., 2017) with the selective inhibitor E3330, we proposed a potentially novel effective mechanism for pain management.

This comparative framework is a significant strength of our work, as it moves beyond isolated docking predictions to contextualize E3330’s potential receptor engagement relative to a well-characterized natural ligand. These results generate a robust hypothesis that E3330 may modulate dopaminergic signaling pathways, which could have important implications for its pharmacological profile and therapeutic applications. This finding offers a starting point for future research into E3330’s mechanism of action, providing novel insights that could enhance understanding of its biological effects and potential clinical utility.

Future directions stemming from this research include clinical trials to assess the safety and efficacy of E3330 in human subjects with inflammatory pain and neurotoxicity. Mechanistic studies are vital to explore the molecular pathways through which E3330 modulates pain signals and mitigates neuroinflammation. Broader applications of E3330 should also be explored to assess its potential in treating other neurodegenerative diseases or conditions linked to oxidative stress and inflammation. Additionally, examining E3330’s protective effects against neurotoxic agents beyond formalin exposure is crucial. Identifying biomarkers for real-time monitoring of E3330’s efficacy could facilitate tailored medicine approaches. Lastly, optimizing formulations for improved delivery and bioavailability, possibly through various administration routes (e.g., oral, intranasal), remains essential to fully harness E3330’s therapeutic potential.

**Figure 11:**
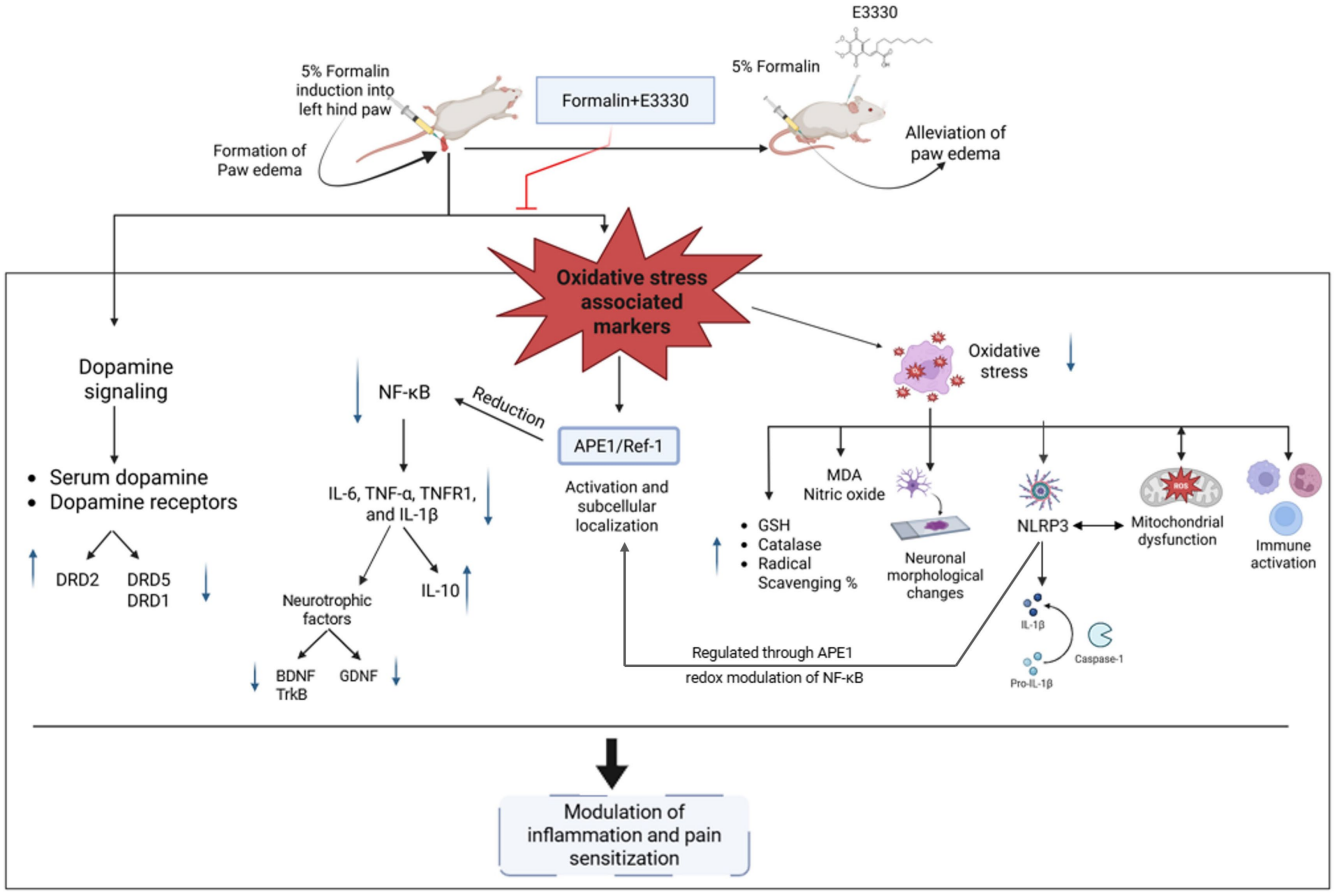
Illustration of the proposed effect of the APE1/Ref-1 redox inhibitor, E3330, in the inflammatory pain condition. Induction of formalin results in increasing the free radicals (ROS). Releasing the free radicals leads to oxidative stress that affects the antioxidant system, inflammasome and causes mitochondrial dysfunction. On the other hand, formalin-induced inflammatory pain is mediated by activation of the oxidative sensor of the cell (APE1/Ref-1) and exhibits a reductive activation of NF-kB and IL-6, leading to upregulate the release of the inflammatory markers, neurotrophic factors and altering the dopaminergic pathway inside the spinal cord (central sensitization). Injection of E3330 showed a promising effect against formalin-induced inflammatory pain by alleviating the oxidative markers and modulating the dopamine receptors inside the spinal cord.

In conclusion, the mechanism by which E3330 regulates the expression of various genes such as MDA, GSH, catalase, NF-κB, IL-6, NLRP3, Caspase-1, IL-1β, iNOS, TNF-*α*, TNFR1, IL-10, BDNF–TrkB, and GDNF likely operates at the intersection of redox modulation and inflammatory signaling pathways, involving a complex interplay of both pro-inflammatory and anti-inflammatory mediators (Figure 11). E3330’s multifaceted actions suggest it orchestrates a coordinated response to oxidative stress and inflammation, potentially providing a comprehensive therapeutic approach to managing inflammatory pain. Further investigations into E3330’s interactions with these mediators could enhance our understanding of its full therapeutic impact.

## 5 Conclusion

This study elucidates the complex interplay between the immune and nervous systems in the context of inflammatory responses resulting from peripheral tissue injury, underscoring the significance of cellular and molecular interactions. Utilizing the formalin model, we gained valuable insights into the nuanced mechanisms of nociception, highlighting the dynamics of both acute and chronic pain. Additionally, our findings regarding the role of E3330 in alleviating inflammatory pain demonstrate its influence on neuroimmune processes and its capacity to modulate neural plasticity, indicating its potential as a therapeutic intervention. The results emphasize the critical need to investigate novel therapeutic targets and strategies for the management of inflammatory pain, with APE1/Ref-1 identified as a particularly promising candidate. This comprehensive analysis paves the way for future research aimed at refining treatment approaches for inflammatory pain, with the overarching goal of achieving therapeutic efficacy while minimizing side effects. Ultimately, these findings contribute to the broader understanding of inflammatory pain mechanisms and offer a foundation for the development of innovative pain management strategies.

## Data Availability

The datasets presented in this study can be found in online repositories. The names of the repository/repositories and accession number(s) can be found below: https://www.ncbi.nlm.nih.gov/ with the accession numbers NM_031144, NM_024148, AF079314.2, M26744, AB250951, L00981, AF329980, NM_031512, X60675, AY176065, NM_019139, NM_012768, NM_012547.2, NM_012546.3, NM_001191642.1, NM_012762.3, and AY265419.1.

## Footnotes

^1^ https://www.uniprot.org/
